# Inhibiting the cytosolic function of CXXC5 accelerates diabetic wound healing by enhancing angiogenesis and skin repair

**DOI:** 10.1038/s12276-023-01064-3

**Published:** 2023-08-01

**Authors:** Eunhwan Kim, Seol Hwa Seo, Yumi Hwang, Yeong Chan Ryu, Heejene Kim, Kyoung-Mi Lee, Jin Woo Lee, Kwang Hwan Park, Kang-Yell Choi

**Affiliations:** 1 Department of Biotechnology, College of Life Science and Biotechnology, Yonsei University, Seoul, South Korea; 2 Department of Orthopedic Surgery, Yonsei University College of Medicine, Seoul, 03722 South Korea; 3 Brain Korea 21 PLUS Project for Medical Science, Yonsei University College of Medicine, Seoul, 03722 South Korea; 4 CK Regeon Inc, Engineering Research Park, 50 Yonsei Ro, Seodaemun-Gu, Seoul, 03722 South Korea

**Keywords:** Diabetes complications, Regenerative medicine, Diabetes complications

## Abstract

Diabetic wound healing, including diabetic foot ulcer (DFU), is a serious complication of diabetes. Considering the complexity of DFU development, the identification of a factor that mediates multiple pathogeneses is important for treatment. In this study, we found that CXXC-type zinc finger protein 5 (CXXC5), a negative regulator of the Wnt/β-catenin pathway, was overexpressed with suppression of the Wnt/β-catenin pathway and its target genes involved in wound healing and angiogenesis in the wound tissues of DFU patients and diabetes-induced model mice. KY19334, a small molecule that activates the Wnt/β-catenin pathway by inhibiting the CXXC5-Dvl interaction, accelerated wound healing in diabetic mice. The enhancement of diabetic wound healing could be achieved by restoring the suppressed Wnt/β-catenin signaling and subsequently inducing its target genes. Moreover, KY19334 induced angiogenesis in hindlimb ischemia model mice. Overall, these findings revealed that restorative activation of Wnt/β-catenin signaling by inhibiting the function of cytosolic CXXC5 could be a therapeutic approach for treating DFUs.

## Diabetes: A road to recovery of long-lasting foot ulcers

Drugs that alleviate a molecular blockade against wound healing and vessel growth could offer effective therapies for diabetic foot ulcers (DFU). Such chronic wounds are a serious consequence of type 2 diabetes, and current therapies only manage the symptoms without providing recovery. Researchers led by Kang-Yell Choi of Yonsei University, Seoul, South Korea, have determined that DFU from human patients and animal models exhibit elevated levels of CXXC5, a protein that inhibits genes associated with healing and angiogenesis. Treatment with an agent that selectively blocks CXXC5 function reversed this inhibition in two different mouse models, accelerating both wound repair and the formation of new blood vessels. The latter is thought to be an especially important aspect of DFU recovery, suggesting that this treatment approach could give long-lasting relief to patients.

## Introduction

Diabetes mellitus (DM) is a chronic disease caused by a glucose metabolism disorder. Accumulated glucose in the blood of DM patients leads to chronic damage to organs and tissues, resulting in serious complications such as retinopathy, nephropathy, neuropathy, and diabetic nonhealing skin ulcers^1,2^.

Diabetic nonhealing skin ulcers, especially diabetic foot ulcers (DFUs), are one of the most serious complications associated with DM. In recent decades, studies of the pathophysiology of DFUs have demonstrated that impaired angiogenesis plays a pivotal role in the development of DFUs by delaying wound healing^3–5^. Angiogenesis, which is the formation of new blood vessels, occurs as early as Day 3 after wounding. Capillary formation in wound tissues provides oxygen and nutrients, especially during the proliferative stage of the wound-healing process. Growth factors such as vascular endothelial growth factor (VEGF) and platelet-derived growth factor (PDGF) are known to be involved in angiogenesis. Among them, VEGF plays unique roles in the multiple stages of wound healing, including re-epithelialization and collagen composition^6^. Current treatments for DFUs mostly focus on using growth factors and antibiotics; however, these treatments only temporarily relieve symptoms and do not result in satisfactory wound healing^7,8^. Therefore, more effective therapeutic agents with new targets are needed.

Accumulating evidence indicates that the Wnt/β-catenin signaling pathway plays an important role in multiple wound healing processes, such as proliferation, regeneration, and angiogenesis^9–11^. The prominent roles of Wnt/β-catenin signaling in the wound healing process may be attributed to the diversity of its target genes involved in wound healing and angiogenesis, including *VEGF*, *keratin 14*, c*ollagen I, Axin2*, and *fibronectin*. For example, transcriptional reduction of VEGF impaired angiogenesis in the wounds of diabetic mice^12^.

In addition, activation of Wnt/β-catenin signaling stimulates wound repair and the regeneration of damaged tissues^11^. Axin2, a well-known target gene of Wnt/β-catenin signaling, is involved in the self-renewal of stem cells in skin tissues^13,14^. We found that Wnt/β-catenin signaling and expression of its target genes are suppressed in the wound tissues of diabetes-induced mice. Therefore, a factor that suppresses Wnt/β-catenin signaling, especially one that drives impaired angiogenesis and wound healing, could be a therapeutic target for diabetic wound healing.

CXXC-type zinc finger protein 5 (CXXC5) acts as a transcription factor in the nucleus and a negative regulator of Wnt/β-catenin signaling within the cytosol. As a transcription factor, CXXC5 is a bone morphogenetic protein 4 (BMP4)‐regulated modulator of neural stem cells and mediates endothelial cell (EC) migration and vessel formation^15–17^. In the cytosol, CXXC5 is a negative regulator of Wnt/β-catenin signaling, and it functions by interacting with the upstream component Dishevelled (Dvl)^18,19^. Cytosolic CXXC5 accumulation has been observed in several diseases, such as osteoporosis, alopecia, and metabolic diseases, and is accompanied by impaired regeneration of damaged tissues^19–23^. Protein transduction domain-fused Dishevelled binding motif (PTD-DBM) peptide, which interferes with the CXXC5-Dvl protein-protein interaction (PPI), promotes cutaneous wound healing and neogenic hair growth through the restoration of Wnt/β-catenin signaling^19,24^. In addition, small molecular mimetics of PTD-DBM peptide that interferes the function of CXXC5, such as KY19382 and KY19334, induce wound healing, hair growth, and longitudinal bone growth, and improve metabolic abnormalities, including diabetes, by activating Wnt/β-catenin signaling^19,20,22–24^. The effectiveness of small molecules that interfere with the function of cytosolic CXXC5 further confirmed the CXXC5-Dvl PPI as a target for treating diseases with suppressed Wnt/β-catenin signaling, such as baldness, bone growth senescence, and metabolic diseases such as obese type 2 diabetes and nonalcoholic steatohepatitis^20–23^.

In this study, we found that cytosolic CXXC5 was highly increased in the dermis layers of the wound tissues of diabetic mice. We also found that the expression levels of wound healing- and angiogenesis-related genes were downregulated, and β-catenin was decreased in diabetic wound tissues. To confirm the role of CXXC5 as a target for treating diabetic wound healing, we treated mice with KY19334 and examined its effects on diabetic wound healing. Topical application of KY19334 markedly accelerated wound healing, increased angiogenesis and enhanced keratin 14 and collagen synthesis. Moreover, we tested the therapeutic effect of KY19334 on impaired angiogenesis in diabetic wounds by using a hindlimb ischemia model. We provide a pathologically significant reverse correlation between the suppression of Wnt/β-catenin signaling and CXXC5 overexpression in DFU patient tissues.

Overall, this study suggests that the inhibiting the function of cytosolic CXXC5 is a potential approach for the treatment of complicated diabetic wounds that requires enhancing wound healing and angiogenesis during tissue regeneration.

## Materials and methods

### Animals

All animal experiments were approved by the Institutional Animal Care and Use Committee of Yonsei University (IACUC-A-201803-713-01, IACUC-A-201806-743-01, IACUC-A-201808-782-01, IACUC-A-201903-873-01, IACUC-A-201904-894-01, IACUC-A- 202105-1261-01, IACUC-A-202207-1505-01, IACUC-A-202210-1560-01).

Two different types of diabetic mouse models were used in this study to investigate diabetic wound healing: HFD+STZ-induced and *db/db*. To generate the HFD+STZ-induced diabetic mouse model, wild-type male C57BL/6 mice (KOATECH) were fed a 60% kcal high-fat diet (HFD; Research Diet, D12492) for 4 weeks. Then, the mice were intraperitoneally injected with streptozocin (STZ; Sigma‒Aldrich, S0130, 40 mg/kg) for 1 week, while control mice were injected with saline. For the *db/db* diabetic mouse model, which has a point mutation in the leptin receptor, 6-week-old male C57BLKS/J-*db/db* diabetic mice (weight, 32–36 g) and age-matched lean C57BLKS/J-*db/m* nondiabetic mice (weight, 16–18 g) were purchased from Japan (SLC) and fed a standard diet (Purina, 38057). *db/db* mice, which develop morbid obesity, pancreatic cell atrophy and chronic hyperglycemia, are widely used to study impaired wound healing in diabetes^25–27^.

To generate diabetic hindlimb ischemia model mice, 4-week-old males were intraperitoneally injected with STZ (100 mg/kg) for 2 days.

*Cxxc5*^*-/-*^ mice were generated as described previously^18^. Heterozygous CXXC5 heterozygous mice were intercrossed for four generations to obtain littermate *Cxxc5*^*+/+*^ and *Cxxc5*^*-/-*^mice and were maintained on a C57BL/6 background. Four‐week‐old male *Cxxc5*^*+/+*^ and *Cxxc5*^*-/-*^ mice were fed a HFD for 4 weeks. Then, the mice were intraperitoneally injected with STZ for 1 week, and the control mice were injected with saline.

To validate the successful establishment of the diabetic model, fasting glucose levels were assessed using a Nocodingone detector (Theragen Etex Co.). After 2 weeks, mice with blood glucose levels higher than 300 mg/dL were considered diabetic and selected for further experiments. All mice were maintained under temperature-controlled and light-controlled (standard 12 h light/12 h dark cycle) conditions and were provided food and water.

### Wound healing model

For the dorsal wound healing model, normal and diabetic mice were anesthetized by an intraperitoneal injection of 2,2,2-tribromoethanol (Sigma‒Aldrich, T48402, 400 mg/kg). After the mice were shaved, 2 full-thickness excisional wounds (diameter = 0.8 cm) were made on the upper back. Then, the mice were randomly separated, and KY19334 (1 mM) or vehicle^28^ was applied topically daily for 16 days. The wounds were photographed, and their sizes were measured periodically with a caliper ruler. Wound-size reduction was calculated using the following equation: wound-size reduction (%) = (At - A0)/A0 × 100, where A0 is the initial wound area and At is the wound area at the indicated time. The mice were sacrificed using CO_2_ on Day 7 to examine vessel formation and on Day 16 for other analyses after wounding, and skin samples were harvested. The undersurface of the skin was photographed to examine newly formed blood vessels, and the blood flow rate was quantified using a laser Doppler perfusion imaging system (Moor LDI 2).

### Hematoxylin and eosin (H&E) staining

The dissected tissues were fixed in 4% neutral paraformaldehyde and embedded in paraffin. The paraffin sections were cut to a thickness of 4 µm. The sections were deparaffinized and rehydrated using gradual ethanol. The sections were stained with hematoxylin for 3 min and with eosin for 1 min. The distance of the wound edges was measured in randomly chosen microscopic areas from 6 independent animal tissues using a Nikon bright-field optical microscope (Nikon TE-2000U). The average wound edge sizes were determined using ImageJ software V1.48 (NIH).

### Collagen staining

The sections were deparaffinized and rehydrated before collagen staining. For picrosirius red staining, tissues were stained with Weigert’s solution for 8 min and stained with picrosirius red solution for 1 h, as previously described^29^.

### Western blot analysis

Tissues were lysed using radioimmunoprecipitation assay (RIPA) buffer (150 mM NaCl, 10 mM Tris, pH 7.2, 0.1% SDS, 1.0% Triton X-100, 1% sodium deoxycholate, and 5 mM EDTA). Equal amounts of whole tissue extracts were separated on 10% SDS polyacrylamide gels and transferred onto nitrocellulose membranes (Protran). Precision Plus Protein Standards (Bio-Rad, 161-0373) were used as protein molecular weight size markers. After being blocked with 7% nonfat dry skim milk for 1 h at room temperature, each membrane was incubated overnight at 4 °C with the following primary antibodies: anti-β-catenin (BD Biosciences, 610154, 1:1000), anti-CXXC5 (Santa Cruz Biotechnology, sc-376348, 1:1000), anti-CD31 (Abcam, ab28364, 1:1000), anti-VEGFR2 (Santa Cruz Biotechnology, sc-6251, 1:500), anti-VEGFA (Abcam, ab52917, 1:500), anti-PCNA (Santa Cruz Biotechnology, sc-56, 1:1000), anti-keratin 14 (Biolegend, 905304, 1:1000), anti-α-SMA (Abcam, ab7817, 1:1000), and anti-total ERK (Cell Signaling Technologies, 9102, 1:1000). Each membrane was then incubated with horseradish peroxidase (HRP)-conjugated anti-rabbit (Bio-Rad, 170-6515, 1:1000) or anti-mouse (Cell Signaling Technology, 7076, 1:1000, 7076) IgG secondary antibodies. Protein bands were visualized using an enhanced chemiluminescence kit (Amersham Pharmacia Biotech), followed by the use of a LAS‐3000 luminescent image analyzer (Fujifilm).

### Immunohistochemical (IHC) analysis

Paraffin sections were deparaffinized and rehydrated. For antigen retrieval, the slides were autoclaved in 10 mM sodium citrate buffer (pH 6.0). The samples were preincubated in phosphate-buffered saline (PBS) and then blocked with 10% bovine serum albumin (BSA) in PBS for 1 h at room temperature.

For fluorescence staining, the samples were incubated overnight at 4 °C with the following primary antibodies: anti-β-catenin (BD Bioscience, 610154, 1:100), anti-β-catenin (Abcam, ab16051, 1:100), anti-CXXC5 (Santa Cruz Biotechnology, sc-376348, 1:100), anti-keratin 14 (Biolegend, 905304, 1:200), anti-PCNA (Santa Cruz Biotechnology, sc-56, 1:100), anti-CD31 (Abcam, ab28364, 1:50), and anti-VEGFA (Abcam, ab52917, 1:100). The slides were washed with PBS, incubated with Alexa Fluor 488- (Thermo Fisher Scientific, A11001, 1:300) or Alexa Fluor 555- conjugated secondary antibodies (Thermo Fisher Scientific, A21428, 1:300) for 1 h at room temperature, and counterstained with DAPI (Sigma‒Aldrich, D9564, 1:5000). Images were captured using an LSM700 META confocal microscope (Carl Zeiss). The fluorescence intensity was quantified using Zen software V3.1 software (Carl Zeiss).

For DAB staining, tissues were incubated with 1% H_2_O_2_ (Samchun Chemicals) for 10 min to block endogenous peroxidase activity. Before the sections were incubated with mouse primary antibodies, mouse IgG was blocked using a mouse-on-mouse (MOM) IgG blocking kit (Vector Laboratories). The sections were incubated overnight at 4 °C with the following primary antibodies: anti-CD31 (Abcam, ab28364, 1:50), anti-VEGFR2 (Santa Cruz Biotechnology, sc-6251, 1:50), anti-VEGFA (Abcam, ab52917, 1:100), and anti-CD34 (Abcam, ab8158, 1:100). After being washed with PBS, the slides were incubated with biotinylated anti-rabbit (Vector Laboratories, BA-1000, 1:300) or biotinylated anti-mouse (Vector Laboratories, BA-9200, 1:300) secondary antibodies for 1 h at room temperature. The samples were stained with DAB (Vector Laboratories, SK-4100) for 5–10 min and counterstained with Mayer’s hematoxylin (Muto Pure Chemicals, 30002). All incubations were conducted in a humidified chamber. Signals were analyzed using a bright-field microscope (Nikon TE-2000U).

### RNA extraction and quantitative real-time PCR

Total RNA was extracted from ground tissue powder and cell lysates using TRIzol reagent (Invitrogen, 15596018) according to the manufacturer’s instructions. Reverse transcription was performed with M-MLV reverse transcriptase (Invitrogen, BRL-28025-013) using 2 µg of total RNA. The synthesized cDNA was diluted to 1 µg/µL. Quantitative PCR was performed on a Rotor-gene Q real-time PCR cycler (Qiagen) using the SYBR Green PCR kit (Qiagen) with the following conditions: 95 °C for 10 min followed by 40 cycles at 95 °C for 5 sec and 60 °C for 15 s. Relative mRNA levels were estimated using the comparative Ct method (∆∆Ct). All mRNA values were normalized to those of *GAPDH*. The primer sequences are listed in Supplementary Table 1.

### Enzyme-linked immunosorbent assay (ELISA)

The levels of secreted VEGF in tissue lysates were analyzed with the Quantikine mouse VEGF ELISA kit (R&D Systems, MMV00) according to the manufacturer’s instructions. The VEGF ELISA kit recognizes both VEGF A and VEGF B.

### Angiogenesis proteome profiler analysis

The expression levels of angiogenesis-related proteins were analyzed using the angiogenesis antibody array kit (R&D Systems, ARY015) according to the manufacturer’s instructions. The blots were developed with enhanced chemiluminescence using a LAS‐3000 luminescent image analyzer (Fujifilm). All data were normalized to the intensity of the reference spots in each membrane according to the manufacturer’s instructions.

### Acute hindlimb ischemia mouse model

After the mice were anesthetized and the hair on the legs was shaved, the femoral artery of the left hindlimb was ligated and removed surgically with a 6–0 PROLENE polypropylene suture (Ethicon Ltd, W8697), while the right limb was not subjected to surgery as a control. The mice were divided, and 150 mL of KY19334 or vehicle was applied daily for 14 days. Blood flow in the ischemic limb was measured on Days 0, 3, 7, and 14 with a laser Doppler perfusion imaging system (Moor LDI 2, Moor Instruments). Perfusion of the ischemic limb was quantified and is presented as a percentage relative to the nonischemic side. After the experiments were completed, the mice were sacrificed, and the gastrocnemius muscles were harvested. The severity of hindlimb ischemia was categorized as limb loss, foot necrosis (death of foot tissues), and limb salvage (similar limb integrity and morphology as normal limb).

### Database

The gene expression profile results are found in NCBI’s GEO database (http://www.ncbi.nlm.nih.gov/geo/) and are accessible through GEO accession number GSE80178.

### Human DFU specimens

The biopsy specimens of paired DFU and non-DFU regions were obtained from patients who had undergone surgery after DFU diagnosis (*n* = 26) between 2021 and 2022 in the Department of Orthopedic Surgery at the College of Medicine of Yonsei University in Seoul, Korea. Experiments using patient samples were approved by the Institutional Review Board of the Clinical Research Institute of Severance Hospital (approval no. 4-2020-0865). Non-DFU regions were defined as skin tissues at a distance of >5 cm from the DFU region. The clinical data of the subjects are presented in Supplementary Table 2.

### Statistical analysis

The data are presented as the means ± standard deviations (SD). Statistical analyses were performed using an unpaired two-tailed Student’s *t* test. Asterisks denote statistically significant differences (^*^P < 0.05; ^**^P < 0.01; ^***^P < 0.001).

## Results

### CXXC5 is overexpressed with the suppression of Wnt/β-catenin signaling in diabetic wounds

To identify the role of CXXC5 in Wnt/β-catenin signaling in the diabetic wound healing process, we used high-fat diet and streptozotocin (HFD+STZ)-induced and *db/db* genetic diabetic model mice to examine the diversity of disease causes^30,31^. Diabetes status was confirmed by measuring the blood glucose levels of HFD+STZ-induced and *db/db* diabetic mice (Supplementary Fig. 1).

**Fig. 1:**
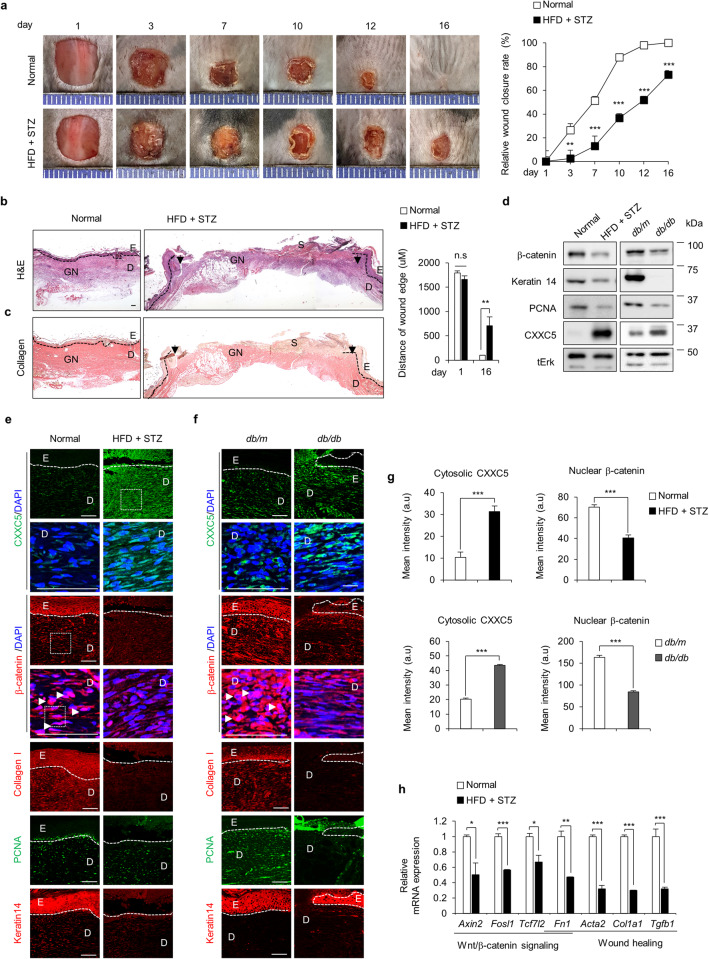
Effects of diabetes on wound healing and CXXC5 and Wnt/β-catenin signaling profiles in mice. Full-thickness wounds (diameter=0.8 cm) were made on the backs of normal and HFD+STZ-induced diabetic mice as described in the Methods. **a** Gross images of the wounds were photographed, and the relative healing rates of the wounds were measured on Days 1, 3, 7, 10, 12, and 16; the results are presented as relative wound closure rates (*n* = 8). **b**, **c** Representative images of H&E and picrosirius red collagen staining are shown. The distances of wound edges were quantified on Days 1 and 16 (*n* = 6). Dashed lines represent the epidermal-dermal boundary. Arrowheads indicate the wound margins; E, epidermis; D, dermis; S, scab; GN, granulation tissue. Scale bars, 100 µm. **d** Western blot analysis of the whole tissue lysate (WTL) of wounds was performed to detect β-catenin, keratin 14, PCNA, CXXC5 and tErk. **e**, **f** Representative images of IHC staining of CXXC5, β-catenin, collagen I, PCNA, and keratin 14 in wound tissues. Dashed lines represent the epidermal-dermal boundary. E, epidermis; D, dermis. **g** Quantitative IHC analysis of cytosolic CXXC5 and nuclear β-catenin, which were examined in the dermal layers of wound tissues (*n* = 6). Scale bars, 100 µm. **h** The relative mRNA expression levels in the wound tissues (*n* = 3). All data are presented as the mean ± SD. *p < 0.05; **p < 0.01; ***p < 0.001 determined by Student’s *t* test.

Full-thickness wounds (diameter = 0.8 cm) were generated on the backs of the shaved mice. Although wound healing was mostly completed by Day 16 days in normal mice, the wounds were not completely healed in HFD+STZ-induced and *db/db* diabetic mice (Fig. 1a and Supplementary Fig. 2a). Therefore, the wound closure rates of the HFD+STZ-induced and *db*/*db* diabetic mice were delayed by 27% and 72%, respectively, compared to those of normal mice (Fig. 1a; right panel and Supplementary Fig. 2a; right panel). Histological analyses showed that the distances between the wound edges were not significantly decreased in either diabetic mouse model (Fig. 1b and Supplementary Fig. 2b). The levels of collagen deposition were decreased in both diabetic mouse models, as determined by picrosirius red staining (Fig. 1c and Supplementary Fig. 2c). The protein levels of CXXC5 were increased and β-catenin was decreased in the wound tissues of both HFD+STZ-induced and *db*/*db* diabetic mice (Fig. 1d). The inverse correlation between CXXC5 and β-catenin, especially their cytosolic and nuclear localizations, respectively, was confirmed by immunohistochemical (IHC) analysis. Similar to β-catenin, the expression levels of collagen I, PCNA, and keratin 14 were decreased in the wound tissues of HFD + STZ-induced and *db/db* diabetic mice (Fig. 1e, f). Quantitative analysis of cytosolic CXXC5 and nuclear β-catenin levels revealed an inverse correlation in diabetic wounds (Fig. 1g). The mRNA expression levels of Wnt/β-catenin target genes, including axin inhibition protein 2 (*Axin2*), Fos-related antigen 1 (*Fosl1*), transcription factor 7-like 2 (*Tcf7l2*), and *fibronectin* (*Fn1*), and tissue component genes, such as alpha-smooth muscle actin (*Acta2*), collagen type 1 alpha 1 (*Col1a1*), and transforming growth factor beta 1 (*Tgfb1*), were reduced in the wound tissues of HFD+STZ-induced diabetic mice (Fig. 1h).

### Angiogenic factors were downregulated in the wounds of diabetic mice

**Fig. 2:**
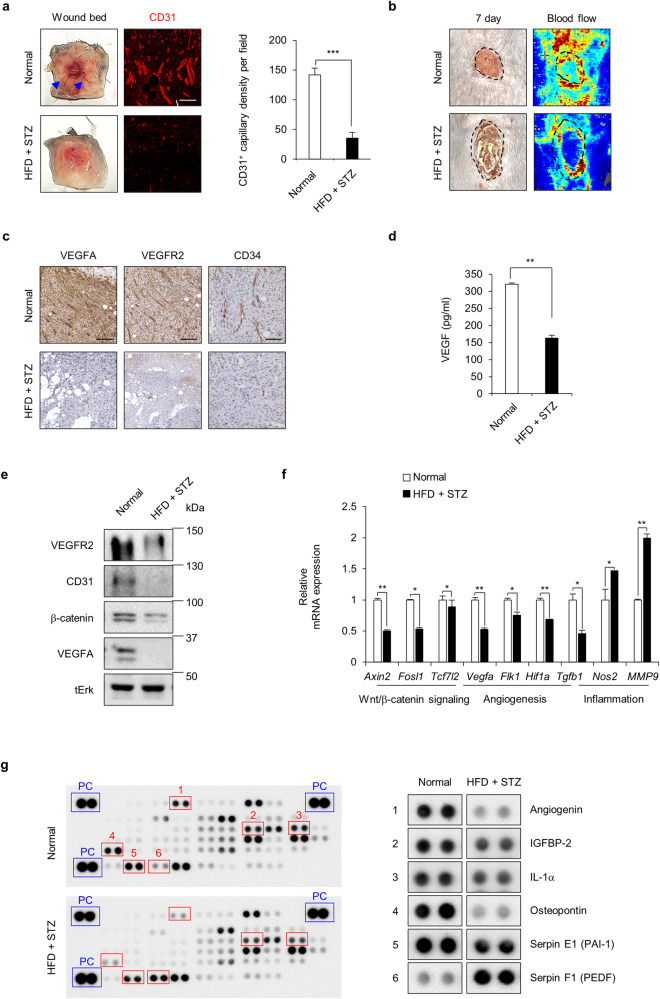
Effects of diabetes on angiogenesis during wound healing in mice. The wound tissues on Day 7 after wounding, which represents the peak of angiogenesis, were subjected to angiogenesis-related analysis. **a** Representative images of wound beds showing the development of capillaries on the subcutaneous surfaces of wound tissues. Blue arrows indicate capillaries. The right panel shows relative CD31^+^ cell numbers in the dermis layers of the wound tissues (*n* = 3). Scale bars, 100 µm. **b** Representative gross images of the wounds and blood flow in the wounds were monitored by the laser Doppler perfusion imaging system. Dashed lines indicate the wound bed boundary. Scale bars, 100 µm. **c** Representative images of DAB staining of VEGFA, VEGFR2, and CD34 in the dermis layers of wound tissues. Scale bars, 100 µm. **d** VEGF concentrations in the WTL of the wounds were measured as described in the Materials and Methods (*n* = 3). **e** Western blot analysis of the WTL of wounds was performed to detect VEGFR2, CD31, β-catenin, VEGFA, and tErk. **f** Relative mRNA expression levels in wound tissues (*n* = 3). **g** Mouse angiogenesis-related proteins in the WTL of wounds were visualized by an antibody array as described in the Materials and Methods. Blue rectangles and red rectangles indicate positive control (PC) and proteins with significantly changed levels, respectively; the right panel shows magnified dot blots. All data are presented as the mean ± SD. *p < 0.05; **p < 0.01; ***p < 0.001 determined by Student’s *t* test.

Because the wound healing rate is largely delayed in diabetes due to impaired angiogenesis^32^, we confirmed the development of capillaries and monitored expression of the endothelial cell marker CD31 in the wound beds at Day 7 after the wounds were made. There were significantly fewer blood vessels on the subcutaneous surface of the diabetic wounds than in normal wounds (Fig. 2a). CD31^+^ capillary density was significantly decreased in the wound sites of HFD+STZ-induced and *db/db* diabetic mice compared with normal mice (Fig. 2a and Supplementary Fig. 3a). Moreover, the ratio of blood flow in the wound tissues of HFD+STZ-induced diabetic mice was lower than that of normal mice (Fig. 2b). The suppression of angiogenesis during wound healing in HFD+STZ-induced and *db/db* diabetic mice was confirmed using 3,3-diaminobenzidine (DAB) staining for the following angiogenesis markers: vascular endothelial growth factor A (VEGFA), vascular endothelial growth factor receptor 2 (VEGFR2), and the endothelial progenitor cell marker CD34 (Fig. 2c and Supplementary Fig. 3b). The inhibition of VEGF signaling correlated with the reduction in VEGF protein levels in the wound tissues of HFD+STZ-induced diabetic mice, as determined by enzyme-linked immunosorbent assay (ELISA) (Fig. 2d).

The decrease in angiogenesis markers in diabetic wounds was confirmed by Western blot analysis, which showed reductions in VEGFR2, CD31, and VEGFA and decreased β-catenin (Fig. 2e). Moreover, the mRNA expression levels of Wnt/β-catenin target genes and angiogenesis-related genes, including *Axin2*, *Fosl1*, *Tcf7l2*, *Vegfa*, *Flk1*, hypoxia-inducible factor 1 alpha (*Hif1a*), and *Tfgb1*, were reduced, whereas inducible nitric oxide synthase (*Nos2*) and inflammatory marker matrix metallopeptidase 9 (*Mmp9*) were elevated in the wound tissues of HFD+STZ-induced diabetic mice (Fig. 2f). Protein chip analysis showed that the decrease in angiogenesis in diabetic mice was further confirmed by a decrease in proangiogenic cytokines such as angiogenin, insulin-like growth factor binding protein 2 (IGFBP-2), interleukin alpha (IL-1α), osteopontin, and serpin E1 (PAI-1) and an increase in the antiangiogenic cytokine serpin F1 (PEDF) in the wound tissue lysates of the diabetic mice (Fig. 2g).

### KY19334 accelerates diabetic wound healing with enhancement of keratin 14 and collagen synthesis

**Fig. 3:**
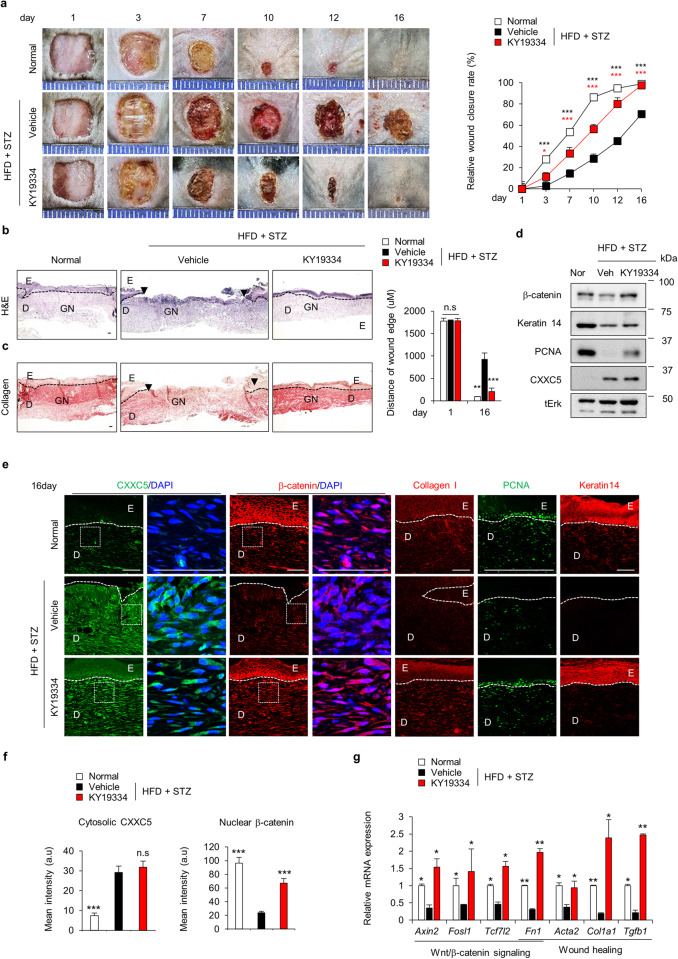
Effect of KY19334 on diabetic wound healing. After the generation of wounds on the backs of normal and HFD+STZ-induced diabetic mice, 1 mM KY19334 or vehicle was applied daily for 16 days. **a** Gross images of wounds were photographed, and the relative healing rates of the wounds were measured on Days 1, 3, 7, 10, 12, and 16; the results are presented as relative wound closure rates (*n* = 8). **b**, **c** Representative images of H&E and picrosirius red collagen staining are shown. Distances of the wound edges were quantified on Days 1 and 16 (*n* = 6). Dashed lines represent the epidermal-dermal boundary. Arrowheads indicate the wound margins; E, epidermis; D, dermis; S, scab; GN, granulation tissue. Scale bars, 100 µm. **d** Western blot analysis of the WTL of wounds was performed to detect β-catenin, keratin 14, PCNA, CXXC5, and tErk. **e** Representative images of IHC staining of CXXC5, β-catenin, collagen I, PCNA, and keratin 14 in wound tissues. Dashed lines represent the epidermal-dermal boundary. E, epidermis; D, dermis. Scale bars, 100 µm. **f** Quantitative IHC analysis of cytosolic CXXC5 and nuclear β-catenin in the dermal layers of the wound tissues (*n* = 6). **g** Relative mRNA expression levels in wound tissues (*n* = 3). All data are presented as the mean ± SD. *p < 0.05; **p < 0.01; ***p < 0.001 determined by Student’s *t* test.

To confirm that the CXXC5-Dvl PPI was a target for the development of therapeutic agents for diabetic wound healing, we tested the effectiveness of KY19334, a small molecule that inhibits the function of cytosolic CXXC5 by interfering with its interaction with Dvl^20,22,23^, on wound healing in diabetic mice. Wound size in KY19334-treated diabetic mice was significantly smaller than that in vehicle-treated diabetic mice at 16 days (Fig. 3a). The rates of wound closure in HFD+STZ-induced diabetic mice were largely improved, and most of the wounds were healed by KY19334, but the wound healing rates decreased by 30% in vehicle-treated diabetic mice (Fig. 3a; right panel).

The rates of wound closure in *db/db* mice were similarly enhanced by KY19334 treatment (Supplementary Fig. 4a). Correlating with the wound closure rates, re-epithelialization and collagen deposition in HFD+STZ-induced and *db/db* diabetic mice were significantly increased by KY19334 treatment (Fig. 3b, c and Supplementary Fig. 4b, c). The suppressed wound healing markers, such as collagen I, PCNA, and keratin 14, in HFD+STZ-induced and *db/db* diabetic mice were restored by KY19334 treatment, as shown by Western blotting and IHC analysis (Fig. 3d–f and Supplementary Fig. 4d). In response to treatment with KY19334, the mRNA expression levels of Wnt/β-catenin target genes, including *Axin2*, *Fosl1*, *Tcf7l2*, *and Fn1*, and those of tissue components, such as *Acta2, Col1a1*, and *Tgfb1*, which were decreased in HFD+STZ-induced diabetic mice, were also restored and were even further increased than those in vehicle-treated mice (Fig. 3g).

### KY19334 restores impaired angiogenesis by recovering the suppressed Wnt/β-catenin signaling pathway

**Fig. 4:**
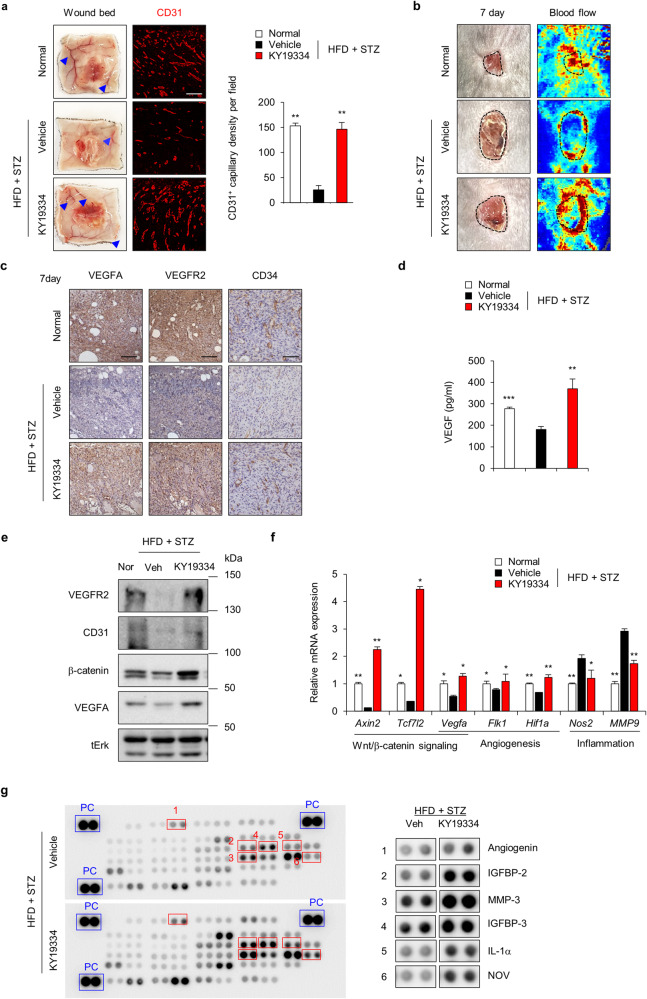
Effect of KY19334 on angiogenesis in diabetic wound healing. After normal and HFD+STZ-induced diabetic mice were wounded and treated with KY19334 for 7 days, wound tissues were harvested to detect angiogenesis-related markers. **a** Representative images of wound beds showing the development of capillaries on the subcutaneous surfaces of the wound tissues. Blue arrows indicate the capillaries. The right panel shows relative CD31^+^ cell numbers in the dermis layers of wound tissues (*n* = 3). Scale bars, 100 µm. **b** Representative gross images of wounds and blood flow in the wounds were monitored with the laser Doppler perfusion imaging system. Dashed lines indicate the wound bed boundary. Scale bars, 100 µm. **c** Representative images of DAB staining of VEGFA, VEGFR2, and CD34 in the dermis layers of wound tissues. Scale bars, 100 µm. **d** VEGF concentrations of the WTL of the wounds (*n* = 3). **e** Western blot analysis of the WTL of wounds was performed to detect VEGFR2, CD31, β-catenin, VEGFA, and tErk. **f** Relative mRNA expression levels in wound tissues (*n* = 3). **g** Mouse angiogenesis-related proteins in the WTL of wounds were visualized by an antibody array according to the manufacturer’s instructions and as described in the Materials and Methods. Blue rectangles and red rectangles indicate the positive control (PC) and the proteins with significantly changed levels, respectively; the right panel shows magnified dot blots. All data are presented as the mean ± SD. *p < 0.05; **p < 0.01; ***p < 0.001 determined by Student’s *t* test.

To confirm whether KY19334 improves diabetic wound healing by enhancing angiogenesis, we examined the effect of KY19334 on blood vessel formation in diabetic wounds. As shown by CD31^+^ capillary density, the reduction in blood vessel formation in the wound beds of HFD+STZ-induced and *db/db* diabetic mice was mostly restored by KY19334 treatment (Fig. 4a and Supplementary Fig. 5a). Moreover, the ratio of blood flow, which was suppressed in the wound tissues of HFD+STZ-induced diabetic mice, was restored by KY19334 treatment (Fig. 4b). Furthermore, the effects of KY19334 on ameliorating the suppression of angiogenesis in diabetic mice were confirmed by measuring angiogenesis markers such as VEGFA, VEGFR2, and CD34 (Fig. 4c and Supplementary Fig. 5b), as well as the production of VEGF (Fig. 4d).

The restoration of angiogenesis in diabetic mice by KY19334 was further confirmed by the restoration of VEGFR2, CD31, and VEGFA expression levels in response to increased β-catenin (Fig. 4e), as shown by Western blot analysis. The effect of KY19334 on the enhancement of angiogenesis via Wnt/β-catenin signaling was further confirmed by measuring the mRNA expression of Wnt/β-catenin target genes, including *Axin2, Tcf7l2*, and *Vegfa*. Angiogenesis-related genes such as *Flk1* and *Hif1a* were increased, whereas inflammatory marker genes, including *Nos2* and *Mmp9*, were decreased (Fig. 4f). The angiogenesis-related cytokines that indicate proangiogenic activity, such as angiogenin, IGFBP-2, MMP-3, IGFBP-3, IL-1α, NOV, and VEGF, were significantly increased by KY19334 treatment, as shown by protein chip analysis of the tissue lysates (Fig. 4g). Overall, KY19334 accelerated diabetic wound healing via the restoration of Wnt/β-catenin signaling, which had been suppressed in diabetic mice.

### Wnt/β-catenin signaling is suppressed by CXXC5 overexpression in diabetic hindlimb ischemia model mice

To provide a more pathologically meaningful model of diabetic wound healing involving angiogenesis, hindlimb ischemia model mice were generated by femoral artery ligation after the induction of STZ-induced diabetes^33^. To evaluate the effect of diabetes on ischemic limb salvage and blood perfusion restoration, the physiological score of ischemic limb status and the recovery of blood flow in the ischemic limb were analyzed. Fourteen days after the induction of ischemia, STZ-induced diabetic mice showed slow restoration of blood flow, and there were more rotten nails or toes in hindlimb ischemia model mice than in normal mice (Supplementary Fig. 6a, b). Diabetic mice had much greater hindlimb ischemic limb loss or necrosis and impaired vessel formation than normal mice (Supplementary Fig. 6c). As shown in Supplementary Fig. 6d, regeneration of the gastrocnemius muscles was decreased in diabetic mice compared to normal mice.

In the ischemic mouse model, angiogenesis directly promoted muscle regeneration and the return of muscle function in the ischemic hindlimb^34^. Therefore, we examined the role of CXXC5 in muscle regeneration. The nuclear expression of β-catenin was significantly reduced with increasing cytosolic CXXC5 expression in the gastrocnemius muscles of diabetic mice (Supplementary Fig. 6e). In the muscles of diabetic mice, the expression of β-catenin was decreased, which correlated with the expression of angiogenesis markers such as CD31 and VEGFA, as well as wound healing markers including PCNA and α-SMA (Supplementary Fig. 6f, g). Overall, angiogenesis and muscle differentiation were decreased with the inactivation of Wnt/β-catenin signaling by cytosolic CXXC5 in diabetic hindlimb ischemia model mice.

### KY19334 restores angiogenic defects in acute hindlimb ischemia diabetic model mice

**Fig. 5:**
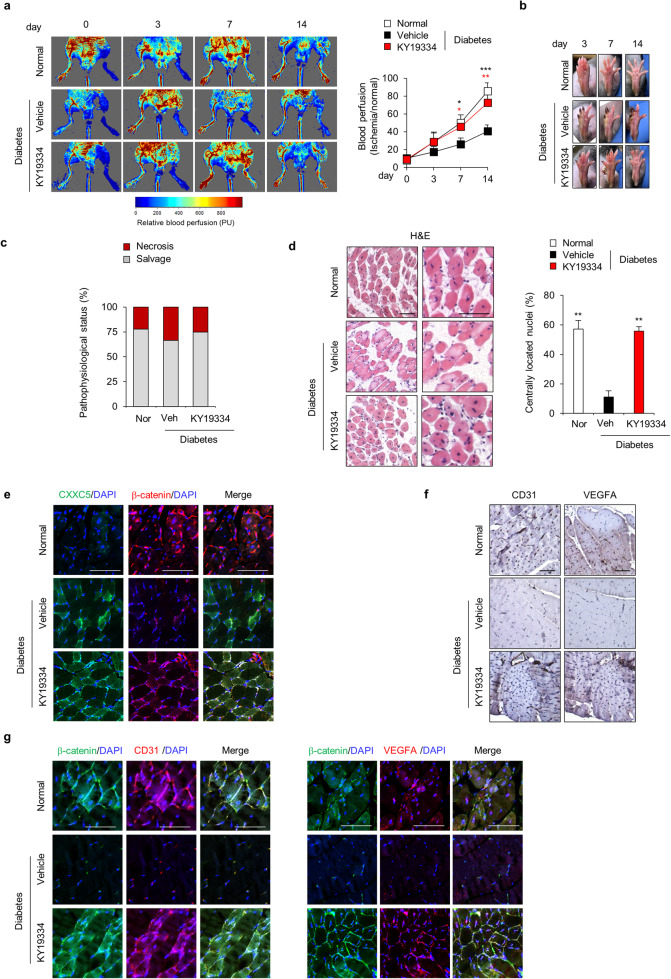
Effects of KY19334 on blood flow recovery in the diabetic hindlimb ischemia model. KY19334 was applied topically every day after the induction of hindlimb ischemia. **a** Representative images of blood reperfusion in the ischemic limb were monitored by the laser Doppler perfusion imaging system. The relative blood reperfusion levels were measured by calculating the perfusion ratio of the ischemic limb to the normal limb (*n* = 6). **b** Representative images of normal and diabetic ischemic limbs with or without KY19334 treatment on Days 3, 7, and 14 after ischemic surgery (*n* = 6). **c** Scoring of physiological status (*n* = 4–6). **d** Representative images of H&E staining of gastrocnemius muscles. Scale bars, 100 µm. **e** Representative images of IHC staining of CXXC5 and β-catenin in gastrocnemius muscles. Scale bars, 100 µm. **f** Representative images of DAB staining of CD31 and VEGFA in gastrocnemius muscles. Scale bars, 100 µm. **g** Representative images of IHC staining of β-catenin, CD31, and VEGFA. Scale bars, 100 µm. All data are presented as the mean ± SD. *p < 0.05; **p < 0.01; ***p < 0.001 determined by Student’s *t* test.

To further validate the recovery of damaged diabetic mouse tissues via Wnt/β-catenin signaling, the effects of KY19334 were tested on diabetic hindlimb ischemia model mice. After treatment with KY19334, the relative blood perfusion rate, which was suppressed in diabetic hindlimb ischemia model mice, was mostly recovered to the level of nondiabetic mice at 14 days (Fig. 5a). After daily topical application of KY19334 for 14 days, rotten toes or nails, which were observed in diabetic hindlimb ischemia, were mostly not observed (Fig. 5b). In addition, hindlimb necrosis was decreased by KY19334 treatment (Fig. 5c). The suppressed regeneration of the gastrocnemius muscle in hindlimb ischemia diabetic mice was mostly restored by KY19334 treatment (Fig. 5d). The recovery of the pathological phenotypes of diabetic hindlimb ischemia by KY19334 correlated with Wnt/β-catenin signaling activation, as revealed by IHC analysis (Fig. 5e). These results also correlated with the improvement in angiogenesis, as shown by the restoration of CD31 and VEGFA expression in diabetic mice, with increased β-catenin (Fig. 5f, g). In summary, treatment with KY19334 improved angiogenesis and muscle regeneration in diabetic hindlimb ischemia.

### The profiles of CXXC5 expression and Wnt/β-catenin signaling in DFU patients correlate with those of diabetic wound healing model mice

**Fig. 6:**
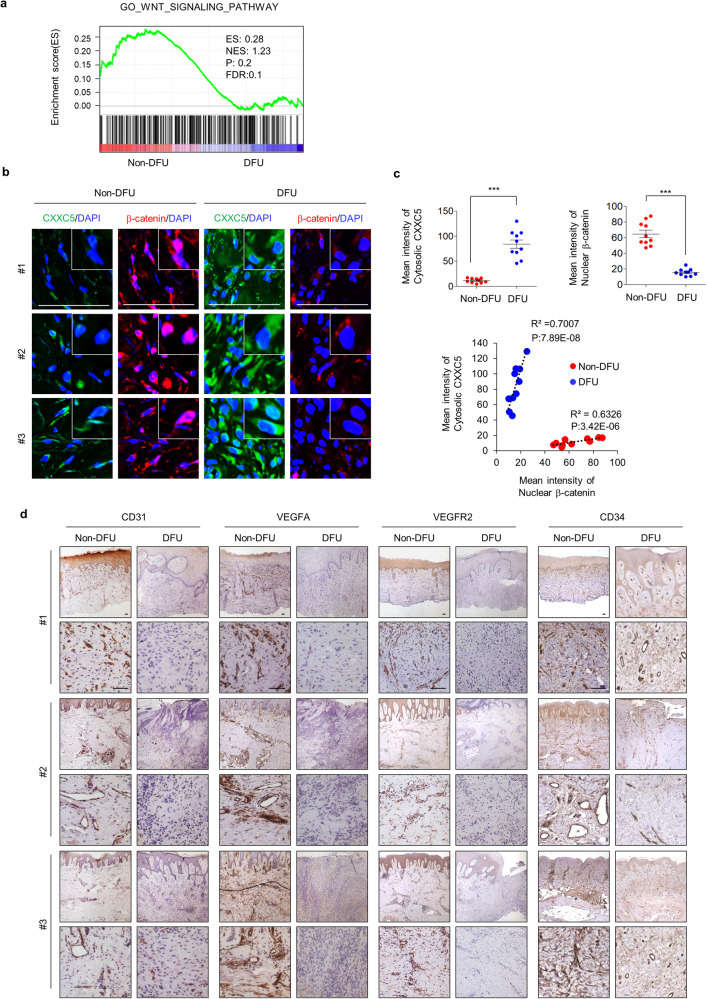
Profiles of CXXC5 and Wnt/β-catenin signaling in the skin tissues of human DFU patients. **a** Gene set enrichment analysis of microarray transcriptome data from patients with DFUs to determine the Wnt/β-catenin signaling-activated gene signature. Black columns indicate 283 enriched genes involving the Wnt/β-catenin signaling pathway in the skin tissues of non-DFU or DFU patients (*n* = 3). ES, enrichment score; NES, normalized enrichment score; FDR, false discovery rate; P: *p-value*. **b**-**d** The skin tissues of non-DFU or DFU regions from patients with DFUs (Supplementary Table 2). **b** Representative IHC images of CXXC5 and β-catenin expression in the dermis layers of skin tissues (*n* = 3). Scale bars, 100 µm. **c** Quantitative mean intensity of IHC staining of cytosolic CXXC5 and nuclear β-catenin was determined and is presented as the correlation of cytosolic CXXC5 with nuclear β-catenin (*n* = 10). **d** Representative images of DAB staining of angiogenesis markers, such as CD31, VEGFA, VEGFR2, and CD34 (*n* = 3). Scale bars, 100 µm. All data are presented as the mean ± SD. *p < 0.05; **p < 0.01; ***p < 0.001 determined by Student’s *t* test.

To elucidate the relationship between Wnt/β-catenin signaling and DFUs, we used gene set enrichment analysis of the non-DFU and DFU regions of patients (Gene Expression Omnibus [GEO]: GSE80178). As shown by microarray analysis, Wnt/β-catenin signaling was downregulated in DFU regions compared to non-DFU regions (Fig. 6a).

To further investigate the status of Wnt/β-catenin signaling related to CXXC5 in DFUs, we obtained biopsy specimens of paired DFU and non-DFU regions from patients who had undergone surgery after DFU diagnosis (*n* = 26). The demographic characteristics, medical status at the screening visit, baseline DFU characteristics, and complications are presented in Supplementary Table 2. The patients were diagnosed with DFUs per standard guidelines, and non-DFU regions were defined as skin tissues at a distance of >5 cm from the DFU regions.

First, DFUs were characterized by incomplete re-epithelialization and collagen deposition in the wound tissues of DFU regions compared with non-DFU regions, as determined by H&E and picrosirius red staining (Supplementary Fig. 7a, b). In the skin tissues of DFU regions, approximately 40% of the patient tissues showed decreased β-catenin and overexpression of CXXC5, especially in the cytosol (Fig. 6b; the data for individual patient images are shown in Supplementary Fig. 8a, b). In this large group of patients, cytosolic CXXC5 and nuclear β-catenin levels were inversely correlated in the skin tissues of the DFU regions compared to non-DFU regions, as observed in the diabetic wound animal models (Fig. 6c). However, no correlation between CXXC5 and nuclear β-catenin levels was observed in the skin tissues of the other group of DFU patients (Supplementary Fig. 8b; lower panel). Angiogenesis was severely suppressed in the dermis layers of DFU regions compared with those of non-DFU regions, as shown by examining CD31, VEGFA, VEGFR2, and CD34 (Fig. 6d and Supplementary Fig. 9). Overall, the wound tissues of 40% of patients with DFUs exhibited suppression of Wnt/β-catenin signaling with CXXC5 overexpression, as observed in diabetic wound healing mouse models.

## Discussion

Diabetic wound healing is a serious complication of diabetes. In particular, DFUs are difficult to treat because of the involvement of various factors in the healing process, including inflammation, proliferation, remodeling, and angiogenesis. Angiogenesis, which supplies oxygen, nutrients, and factors required for the wound healing process, is essential for the recovery of DFUs. The current treatments for DFUs are mostly growth factor-based therapies, but these have limitations due to temporal effectiveness and poor efficacies with limited capability for skin-tissue regeneration in wound healing.

Signaling pathways such as the transforming growth factor beta (TGF-β) pathway, the Notch pathway, the Hedgehog pathway, and the Wnt/β-catenin pathway play important roles in the regeneration of skin^35^. Among these pathways, the Wnt/β-catenin signaling pathway plays important roles in tissue regeneration and the regulation of adult stem cells during wound healing^9^. Moreover, the Wnt/β-catenin pathway is important for angiogenesis, and proteins involved in angiogenesis, such as VEGFA, BMP4, and VEGFR2, are direct and indirect transcriptional targets^12,36^. Therefore, the Wnt/β-catenin pathway could be an ideal target for the development of drugs to treat diabetic wounds, especially DFUs, which are accompanied by severe angiogenic defects.

In this study, we found that Wnt/β-catenin signaling was suppressed in the wounds of patients with DFUs, as well as in the wounds of diabetic model mice induced by chemical and genetic approaches, as indicated by the significant reduction in nuclear active β-catenin. Moreover, we found that the overexpression of CXXC5, a negative regulator of the Wnt/β-catenin signaling pathway that functions through Dvl interactions, is a major cause of DFUs, as indicated by the histological analysis of skin tissues from both DFU patients and diabetic model mice. The potential use of CXXC5 as a target for diabetic wound healing, especially DFUs, was indicated by significant improvements in wound healing rates in HFD+STZ-induced and *db/db* diabetic mice and diabetic hindlimb ischemia model mice treated with KY19334, a functional inhibitor of CXXC5. The suppression of Wnt/β-catenin signaling via the overexpression of CXXC5 in the wound tissues of DFU patients, as well as the inverse correlation between nuclear active β-catenin and cytosolic CXXC5, supports the idea of the CXXC5-Dvl PPI as a pathologically meaningful target for diabetic wounds and DFUs. The wound healing enhancement in diabetic mice caused by KY19334 could be achieved by restoring the suppressed Wnt/β-catenin signaling and subsequent recovery of its target genes encoding factors required for angiogenesis, such as CD31, VEGFA, and VEGFR2, and wound healing markers such as collagen I, keratin 14, α-SMA, and PCNA.

Although we previously observed that nondiabetic normal wound healing was significantly accelerated in *Cxxc5*-knockout mice^19^, we did not observe healing enhancement in diabetes-induced *Cxxc5*-knockout mice (Supplementary Fig. 10). These unexpected results in *Cxxc5*-knockout mice treated with the functional CXXC5 inhibitor, KY19334, indicate that CXXC5 may play other roles in diabetic wound healing that differ from those involved in wound healing in normal mice. CXXC5 has different roles depending on its subcellular localization. For example, in the nucleus, CXXC5 serves as a transcriptional activator of *Flk-1*, which encodes VEGFR2^16^, whereas cytosolic CXXC5 plays a role in the inactivation of Wnt/β-catenin signaling. Knocking out *Cxxc5* abolishes both effects of CXXC5, which is a transcriptional activator mediating endothelial differentiation and vessel formation and a negative regulator of Wnt/β-catenin signaling, which results in delayed diabetic wound healing (Supplementary Fig. 11a). The lack of enhanced wound healing in diabetic *Cxxc5*-knockout mice may be due to the abolishment of the role of CXXC5 as a transcriptional activator of *Flk-1*, which encodes the angiogenic factor VEGFR2 (Supplementary Fig. 11a vs. b). These results further suggest the critical role of angiogenesis in diabetic wound healing. Unlike the *Cxxc5*-knockout mice, KY19334, which specifically abrogates the function of cytosolic CXXC5, enhanced diabetic wound healing by restoring suppressed Wnt/β-catenin signaling, inducing VEGF, BMP4, collagen I, fibronectin, and transcriptional activation of *Flk-1*, which encodes VEGFR2 (Supplementary Fig. 11c).

In this study, the effectiveness of the chemical CXXC5-Dvl PPI inhibitor on diabetic wound healing, including a diabetic hindlimb ischemia mouse model, suggests that compounds such as KY19334 can be developed as wound healing agents for the treatment of diabetic wounds. Overall, restoration of the suppressed Wnt/β-catenin signaling by specific interference of the CXXC5-Dvl PPI could be a potential therapeutic approach for diabetic wound healing, as highlighted by DFUs.

## Supplementary information

Supplementary Information
