# Supplementary material for: Inhibiting the cytosolic function of CXXC5 accelerates diabetic wound healing by enhancing angiogenesis and skin repair
Source: Exp Mol Med. 2023 Aug 1;55(8):1770–82. doi: 10.1038/s12276-023-01064-3 (PMC10474114; doi:10.1038/s12276-023-01064-3)
Supplement: Supplementary file 1 — Supplementary Information [file 12276_2023_1064_MOESM1_ESM.pdf]

Inhibiting the cytosolic function of CXXC5 accelerates diabetic wound healing by enhancing angiogenesis and skin repair

Eunhwan Kim[1], Seol Hwa Seo[1], Yumi Hwang[1], Young Chan Ryu[1], Heejene Kim[1], Kyoung-Mi Lee[2], Jin Woo Lee[2,3], Kwang Hwan Park[2]✉, Kang-Yell Choi[1,4] ✉

[1]Department of Biotechnology, College of Life Science and Biotechnology, Yonsei University, Seoul, South Korea. [2]Department of Orthopedic Surgery, Yonsei University College of Medicine, Seoul 03722, South Korea. [3]Brain Korea 21 PLUS Project for Medical Science, Yonsei University College of Medicine, Seoul 03722, South Korea. [4]CK Regeon Inc, Engineering Research Park, 50 Yonsei Ro, Seodaemun-Gu, Seoul 03722, South Korea. ✉email: khpark@yuhs.ac, kychoi@yonsei.ac.kr

Supplementary Fig. 1

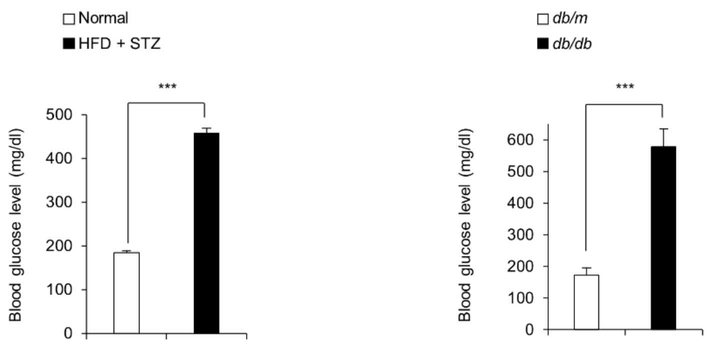

Supplementary Fig. 1 Blood glucose levels of HFD + STZ-induced and *db/db* diabetic mice. Total blood of mice was collected by tail vein before the excision of the wounds (n = 8). Blood glucose levels of normal mice and HFD + STZ-induced and *db/db* diabetic mice were measured as described in the Materials and method section. All data are presented as the mean ± SD. *p < 0.05; **p < 0.01; ***p < 0.001 determined by student's *t* test.

Supplementary Fig. 2

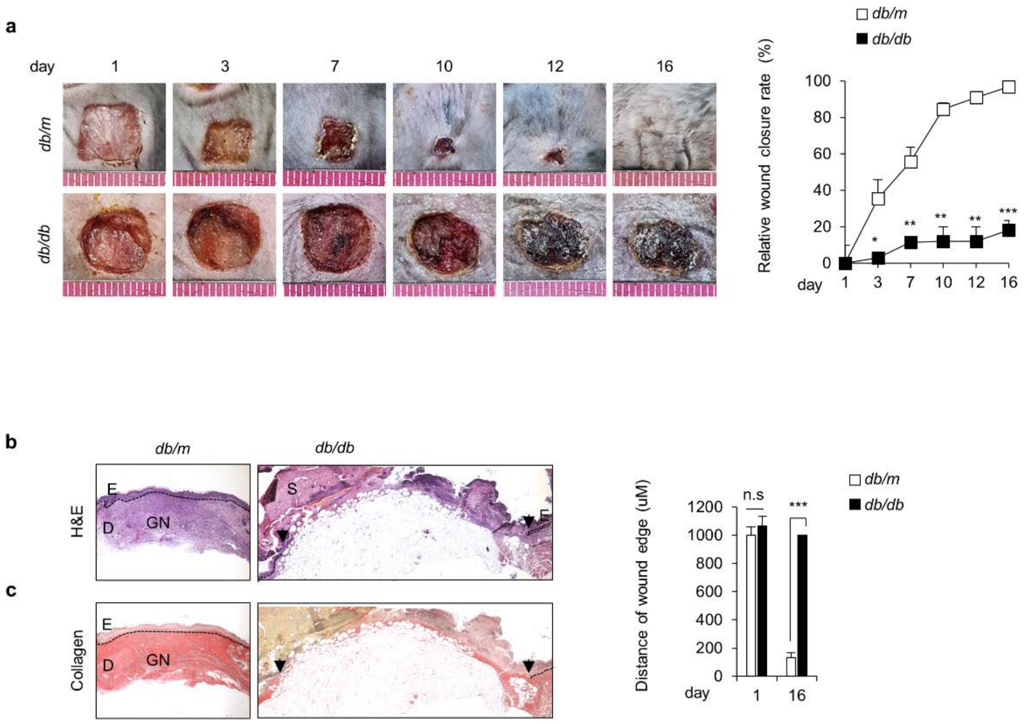

Supplementary Fig. 2 Delayed wound healing in *db/db* diabetic mice. a Gross images of wounds were photographed and the relative healing rates of wounds were measured on days 1, 3, 7, 10, 12 and 16 after wounds were made, respectively and presents as relative wound closure rates (n = 4). **b, c** Representative images of H&E and picrosirius red collagen staining are shown. Distances of the wound edges were quantified on days 1 and 16 (n = 6). Dashed lines represent the epidermal–dermal boundary. Arrowheads indicate the wound margins; E, epidermis; D, dermis; S, scab; GN, granulation tissue. Scale bars, 100 µm. All data are presented as the mean ± SD. *p < 0.05; **p < 0.01; ***p < 0.001 determined by student’s *t* test.

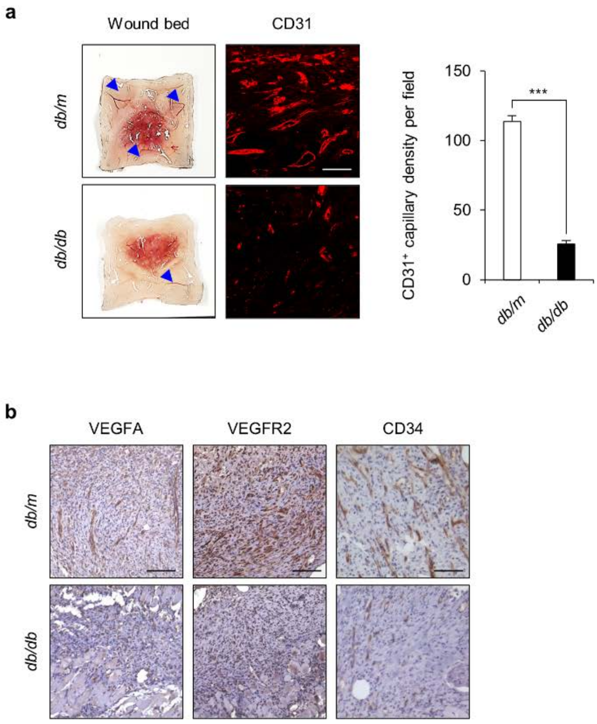

Supplementary Fig. 3 Impaired angiogenesis in wound tissues from *db/db* diabetic mice. **a** Representative images of wound beds showing the development of capillaries on the subcutaneous surface of wound tissues. Blue arrows indicate the capillaries. The right panel shows the relative $CD31^+$ capillary density in the dermis layers of the wound tissues (n = 3). Scale bars, 100 µm. **b** Representative images of DAB staining for VEGFA, VEGFR2, and CD34 in the dermis layers of the wounds. Scale bars, 100 µm. All data are presented as the mean ± SD. *p < 0.05; **p < 0.01; ***p < 0.001 determined by student's *t* test.

Supplementary Fig. 4

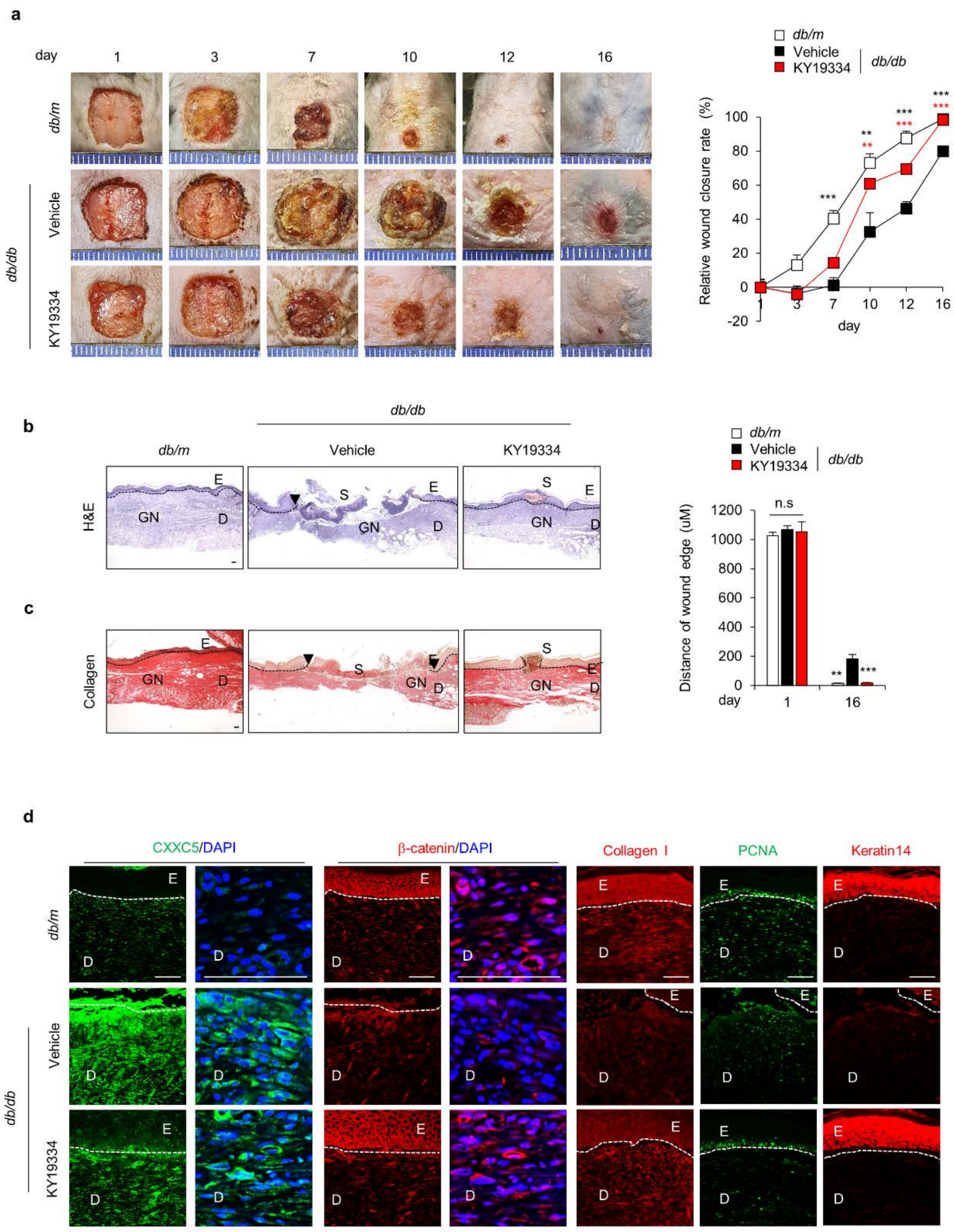

Supplementary Fig. 4 Acceleration of wound healing by KY19334 treatment in *db/db* diabetic mice. a Gross images of wounds were photographed and the relative healing rates of wounds were measured on days 1, 3, 7, 10, 12, and 16 after the wounds were made, presented as relative wound closure rates (n = 6). **b, c** Representative images of H&E and picrosirius red collagen staining are shown. Distances of the wound edges were quantified on days 1 and 16 (n = 6). Dashed lines represent the epidermal–dermal boundary. Arrowheads indicate the wound margins; E, epidermis; D, dermis; S, scab; GN, granulation tissue. Scale bars, 100 μm. **d** Representative images of IHC staining for CXXC5, β-catenin, collagen I, PCNA, and keratin 14 in the wound tissues. Dashed lines represent the epidermal–dermal boundary. E, epidermis; D, dermis. Scale bars, 100 μm. All data are presented as the mean ± SD. *$p < 0.05$; **$p < 0.01$; ***$p < 0.001$ determined by student's *t* test.

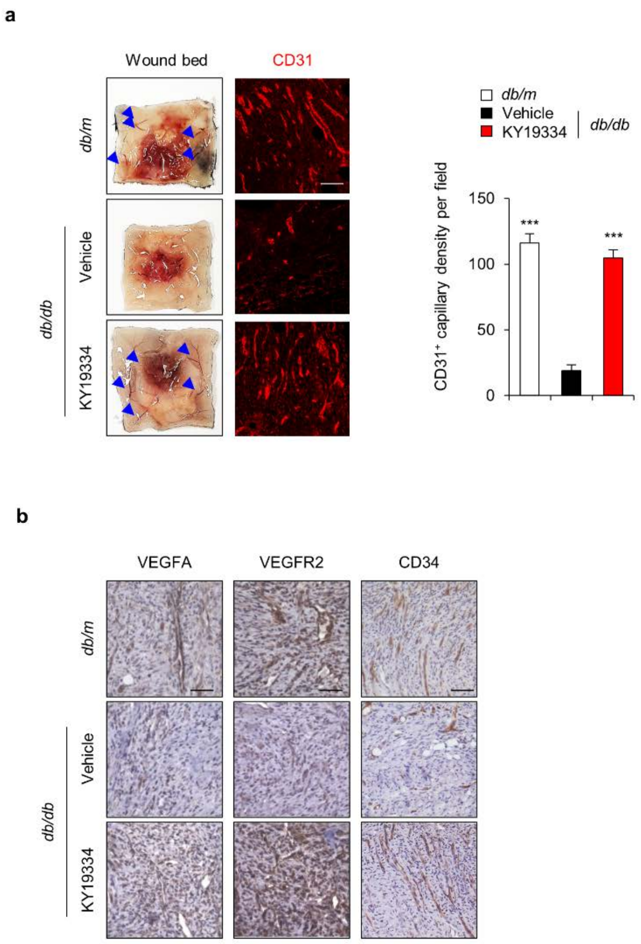

Supplementary Fig. 5 Improvement of angiogenesis by KY19334 treatment in *db/db* diabetic mice. a Representative images of wound beds showing the development of capillaries on the subcutaneous surfaces of the wound tissues. Blue arrows indicate the capillaries. Scale bars, 100 µm. The right panel reveals relative $CD31^+$ capillary density in the dermis layers of the wound tissues (n = 3). Scale bars, 100 µm. **b** Representative images of DAB staining for VEGFA, VEGFR2, and CD34 in the dermis layers of the wound tissues, Scale bars, 100 µm. All data are presented as the mean ± SD. *p < 0.05; **p < 0.01; ***p < 0.001 determined by student's *t* test.

Supplementary Fig. 6

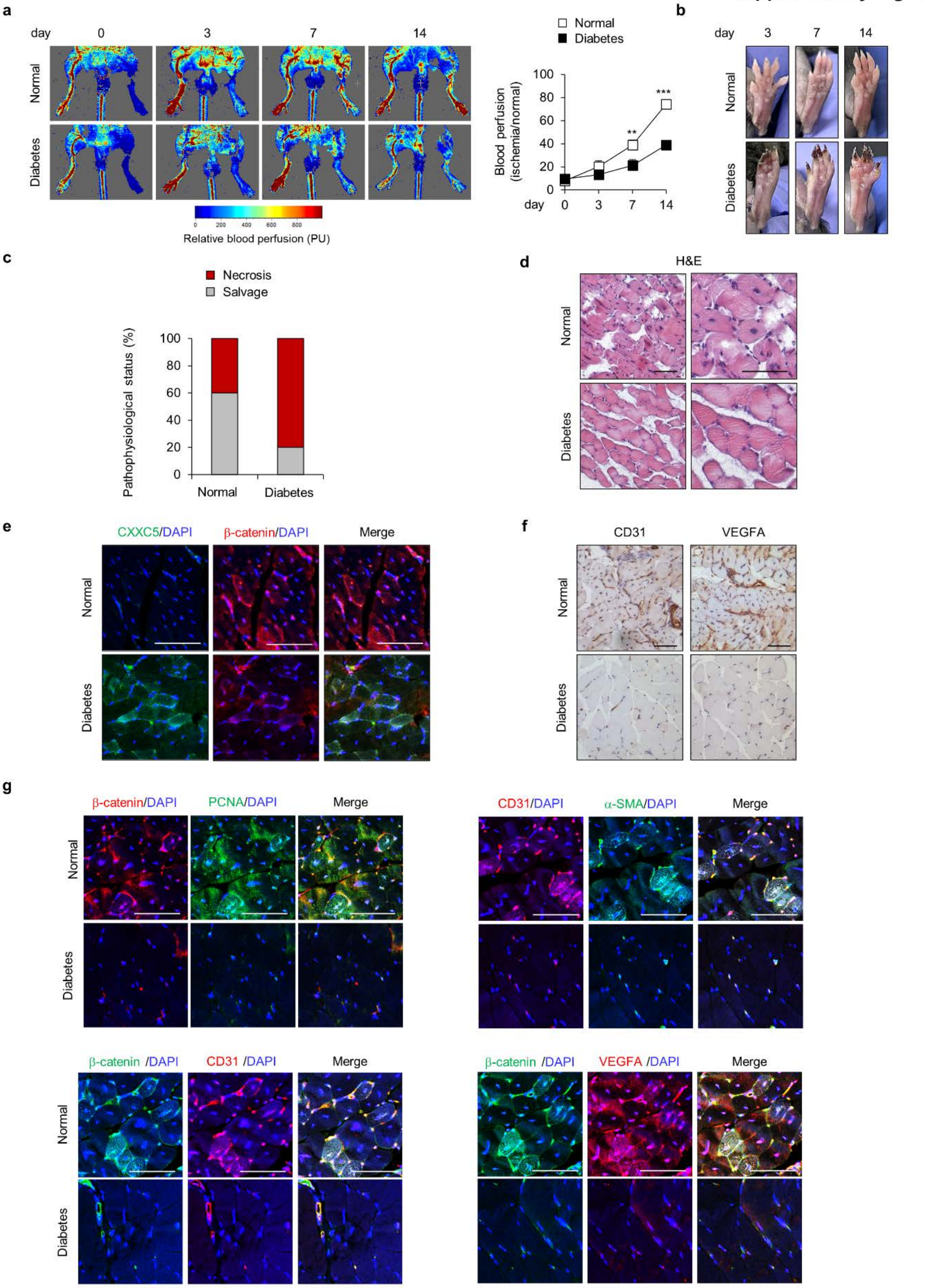

Supplementary Fig. 6 Effects of diabetes on impaired blood flow recovery in the hindlimb ischemia model. The acute diabetic hindlimb ischemia model mice were generated by ligation and removal of the proximal and distal sites of the femoral artery of the left hindlimb after induction of STZ-induced diabetic mice, as described in the Materials and methods section. **a** Representative images of blood reperfusion in the ischemic limb were monitored by the laser doppler perfusion imaging system. The relative blood reperfusion levels were measured by calculation of the perfusion ratio of the ischemic limb to the normal limb on days 0, 3, 7, and 14 after ischemic injury (n = 6). **b** Representative images were photographed on days 3, 7, and 14 after ischemic surgery. **c** Scoring of physiological status. (n = 3 – 5). **d** Representative images of H&E staining in the gastrocnemius muscles. Scale bars, 100 µm. **e** Representative images of IHC staining for CXXC5 and β-catenin in the gastrocnemius muscle. Scale bars, 100 µm. **f** Representative images of DAB staining for CD31 and VEGFA in the gastrocnemius muscles. Scale bars, 100 µm. **g** Representative images of IHC staining for β-catenin, PCNA, CD31, α-SMA, and VEGFA. Scale bars, 100 µm. All data are presented as the mean ± SD. *$p < 0.05$; **$p < 0.01$; ***$p < 0.001$ determined by student's *t* test.

Supplementary Fig. 7

a

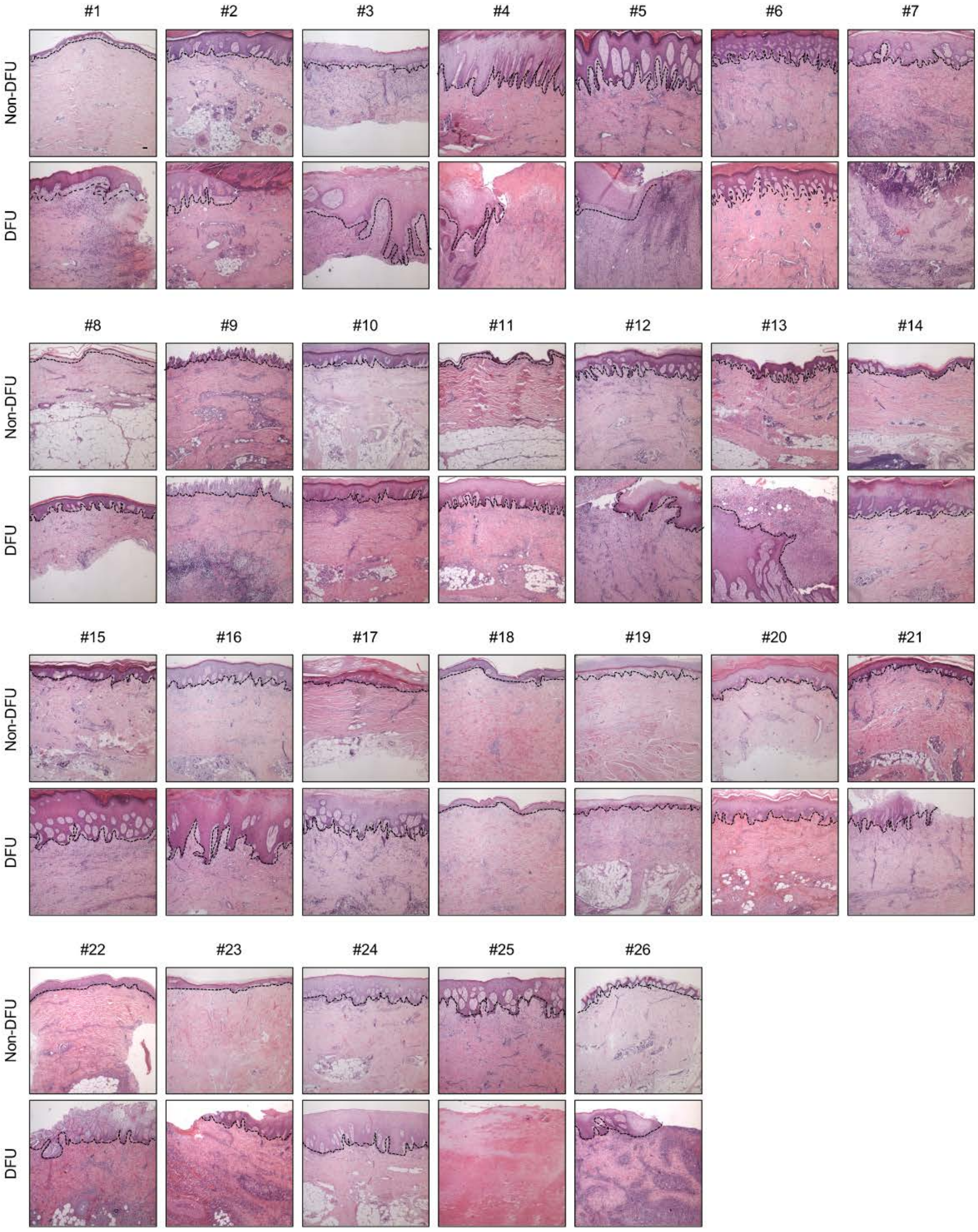

Supplementary Fig. 7

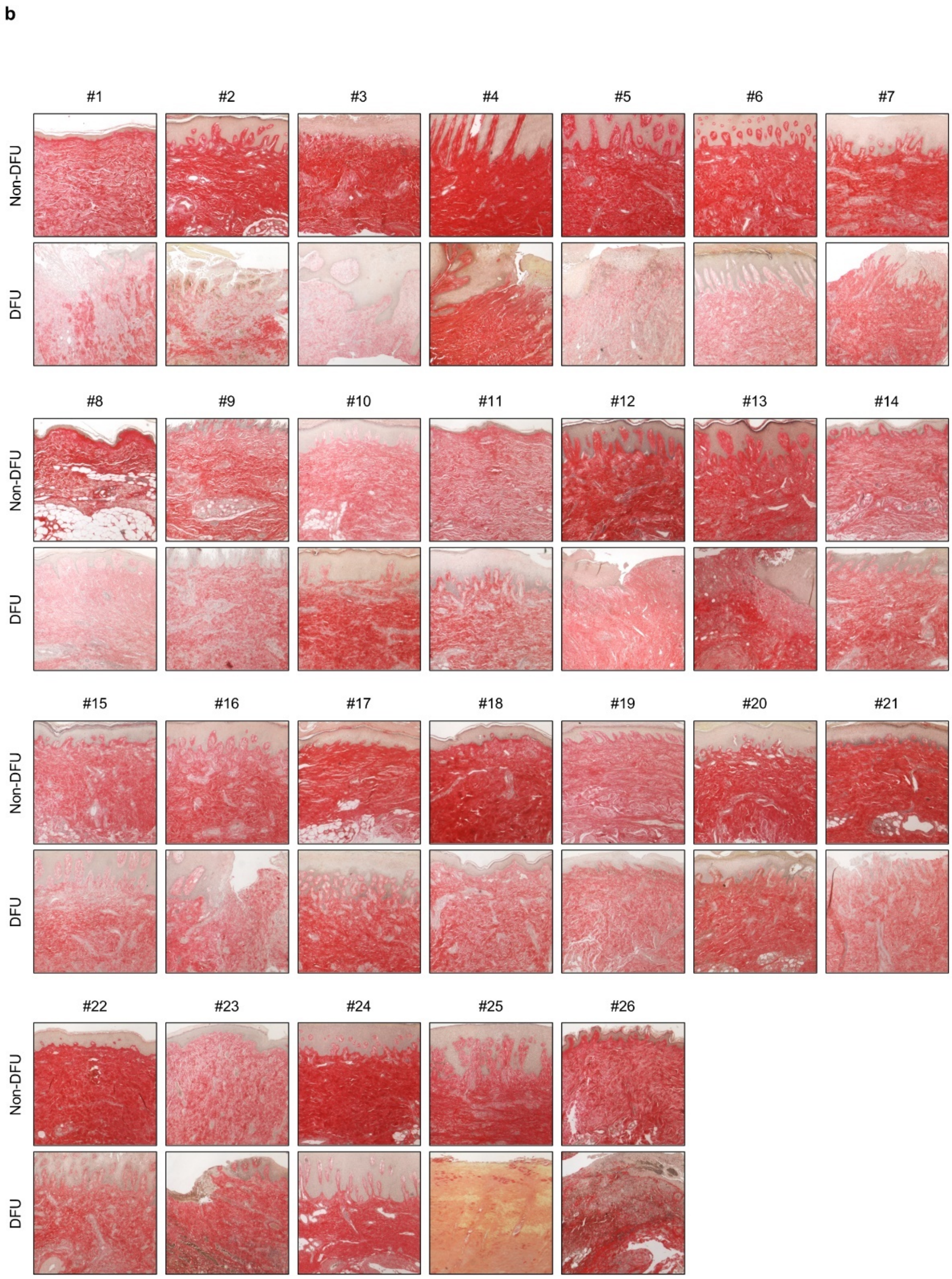

Supplementary Fig. 7 Histological analyses in the skin tissues of DFU patients. Representative images of **a** H&E staining **b** and picrosirius red collagen staining in the skin tissues of non-DFU or DFU regions from patients with DFUs. Scale bars, 100 µm.

Supplementary Fig. 8

a

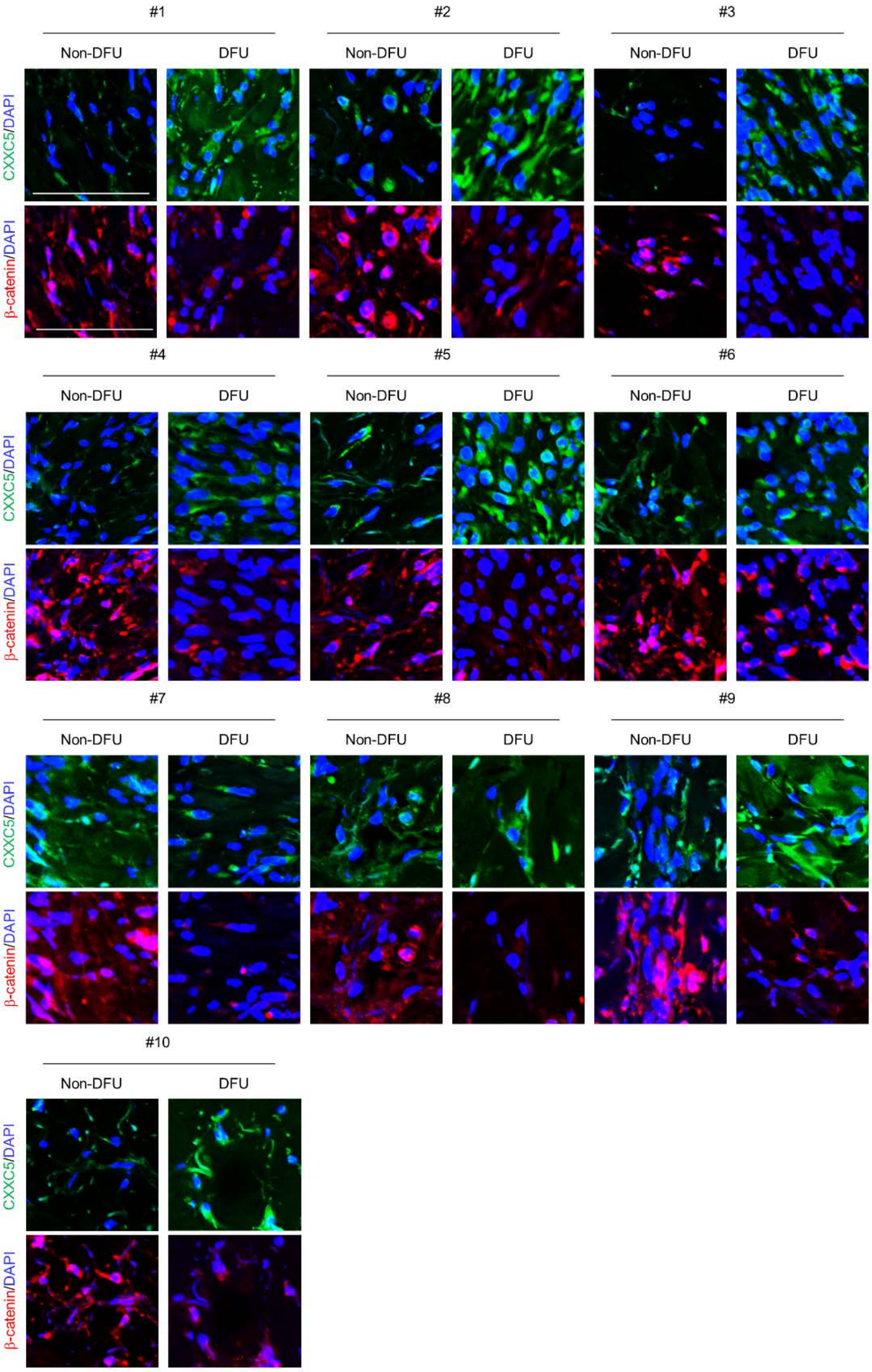

Supplementary Fig. 8

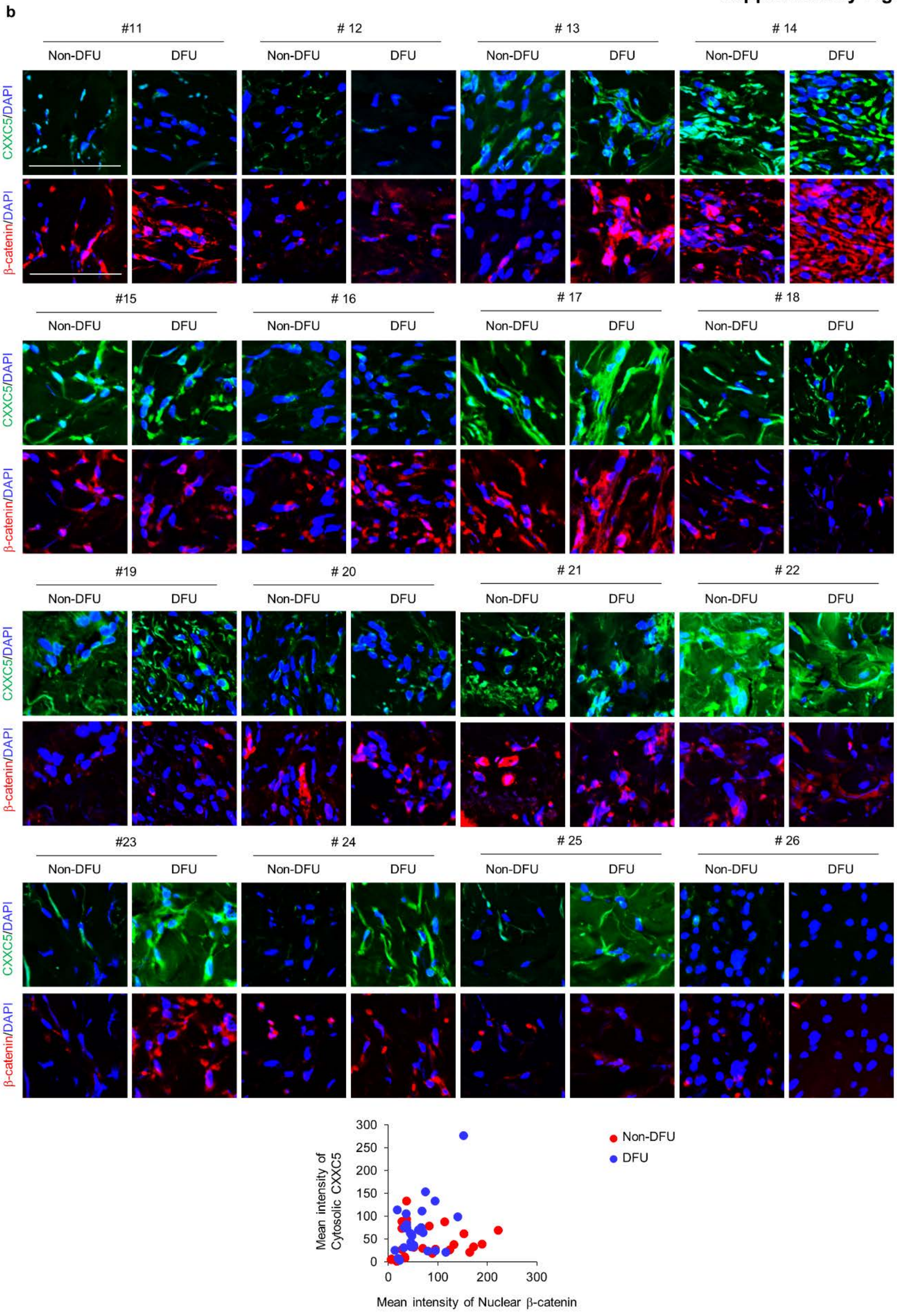

Supplementary Fig. 8 Profiles of CXXC5 and β-catenin expressions in the skin tissues of non-DFU or DFU regions from patients with DFUs. Representative IHC images of CXXC5 and β-catenin expression in the skin tissues of non-DFU or DFU regions from patients with DFU. (n = 26). **a** In about 40% of the tissues of all DFU patients, CXXC5 and β-catenin expression showed an inverse correlation. (n = 10). Scale bars, 100 μm. **b** Representative IHC images of CXXC5 and β-catenin in the remaining DFU patient tissues. The quantitative mean intensity values of the IHC staining for cytosolic CXXC5 and nuclear β-catenin did not show a correlation between cytosolic CXXC5 and nuclear β-catenin. (n = 16). Scale bars, 100 μm.

Supplementary Fig. 9

a

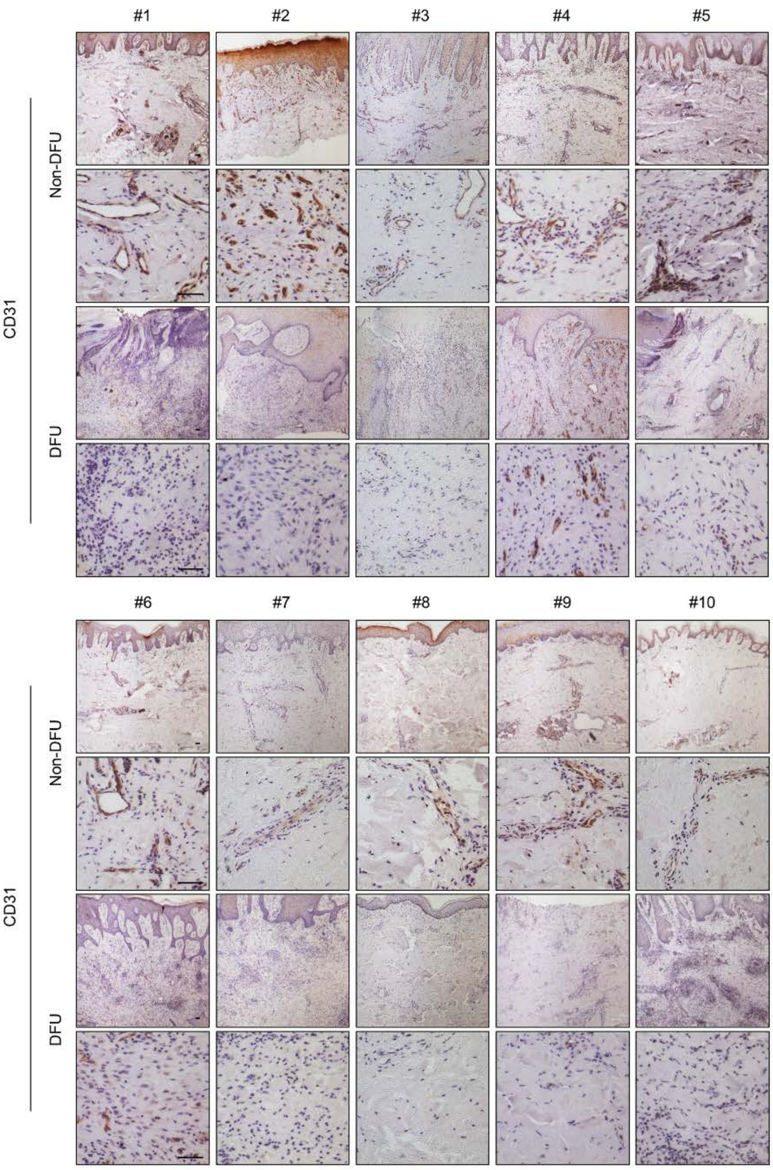

Supplementary Fig. 9

b

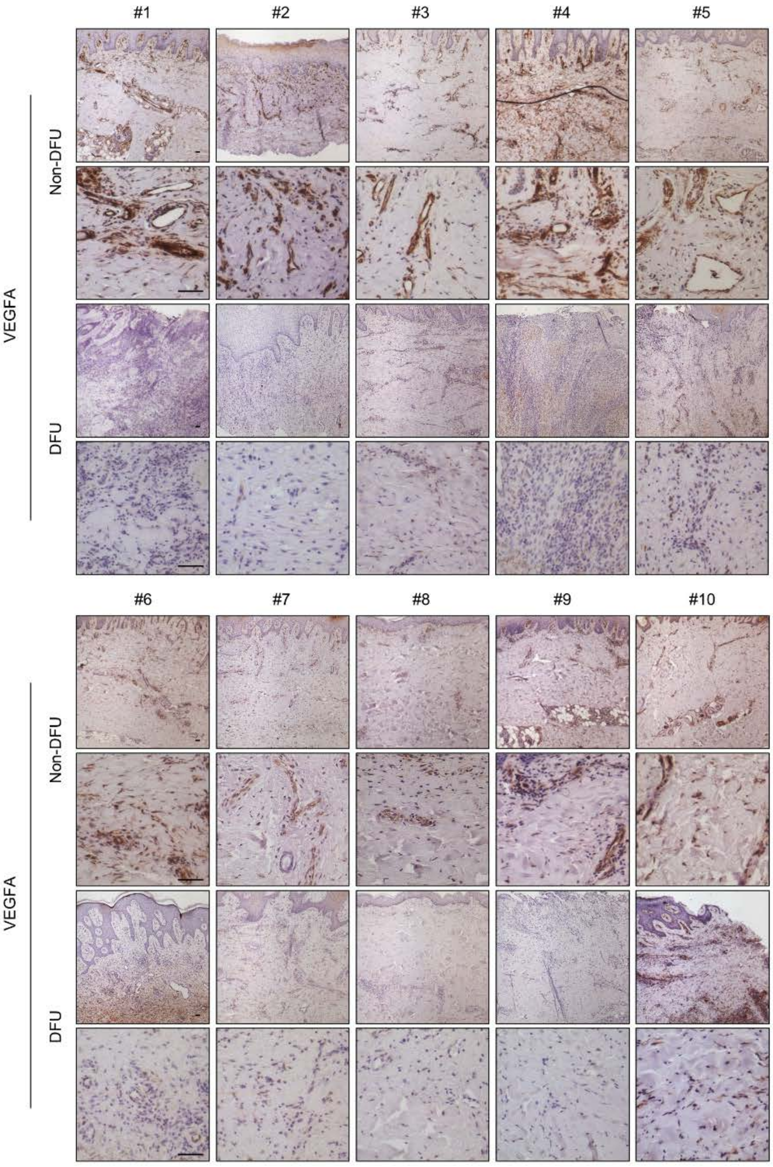

c

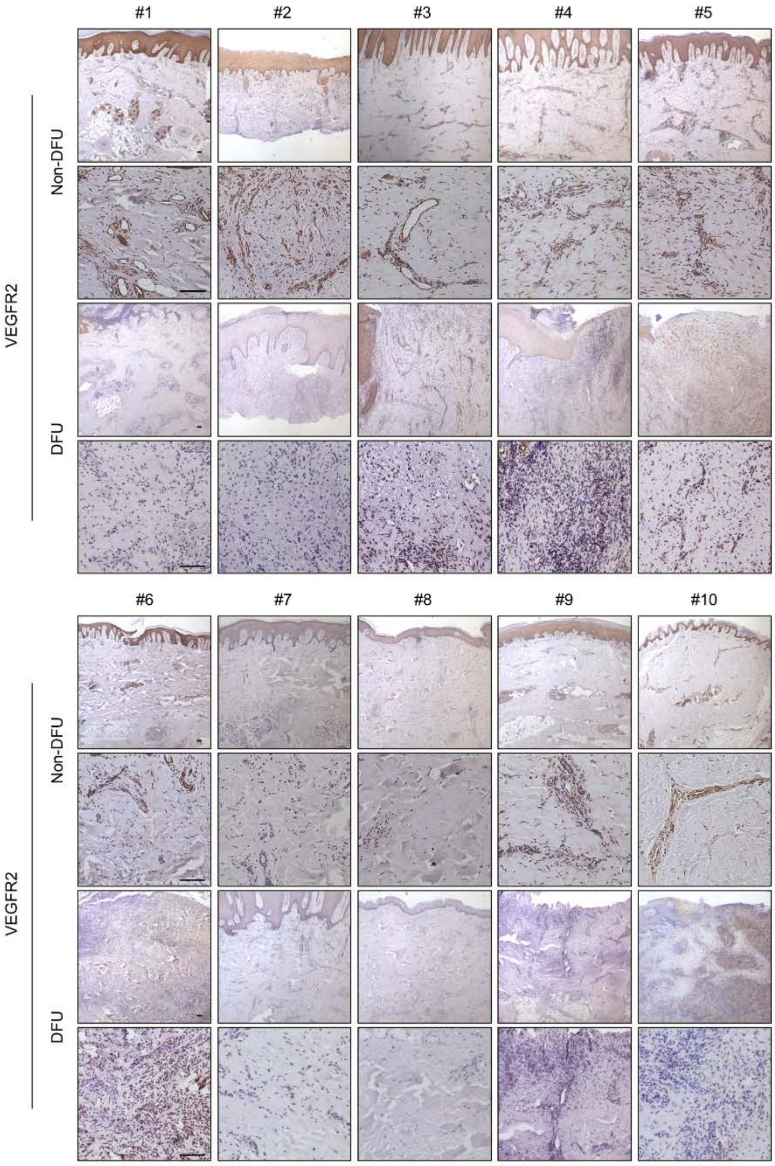

Supplementary Fig. 9 Angiogenesis was decreased in the skin tissues from DFU patients. Representative images for **a** CD31, **b** VEGFA, and **c** VEGFR2 as detected by DAB staining of the dermis layers of the skin tissues from non-DFU or DFU regions of DFU patients. (n = 10). Scale bars, 100 µm.

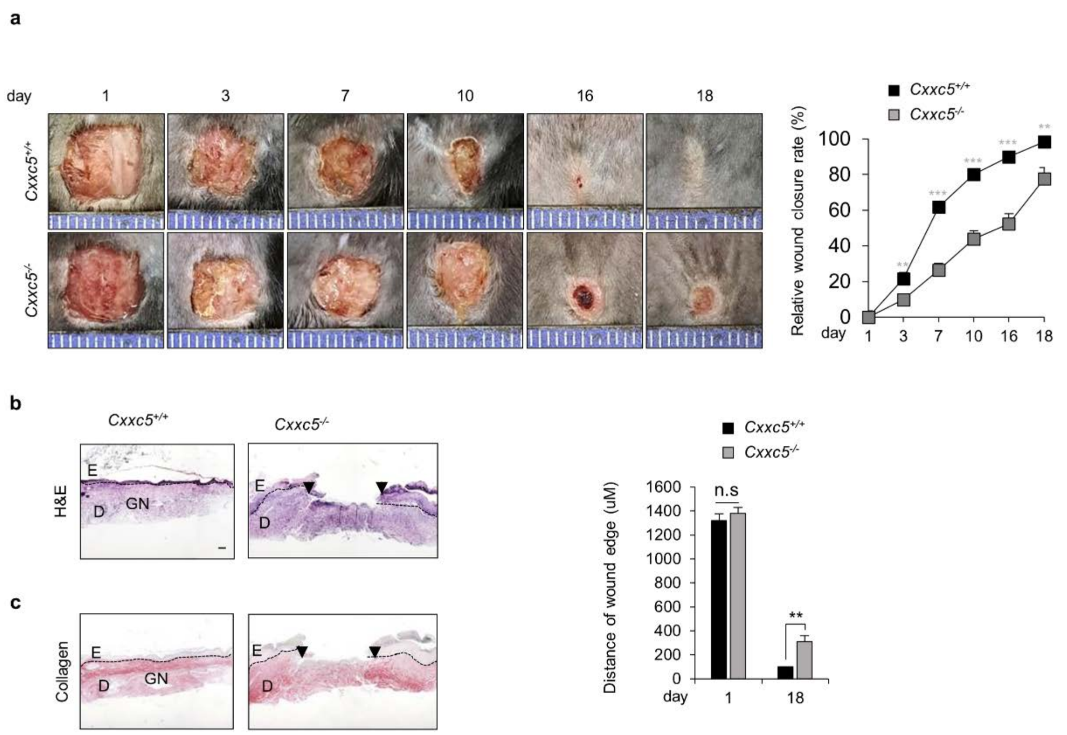

Supplementary Fig. 10 Effects of diabetes on wound healing in *Cxxc5*$^{+/+}$ and *Cxxc5*$^{-/-}$ mice.

a Gross images of wounds were photographed and the relative healing rates of wounds were measured on days 1, 3, 7, 10, 16, and 18 after the wounds were made, presented as relative wound closure rates (n = 3). **b, c** Representative images of H&E and picrosirius red collagen staining are shown. Distances of the wound edges were quantified on days 1 and 18 (n = 3). Dashed lines represent the epidermal–dermal boundary. Arrowheads indicate the wound margins; E, epidermis; D, dermis; S, scab; GN, granulation tissue. Scale bars, 100 µm. All data are presented as the mean ± SD. *p < 0.05; **p < 0.01; ***p < 0.001 determined by student's *t* test.

Supplementary Fig. 11

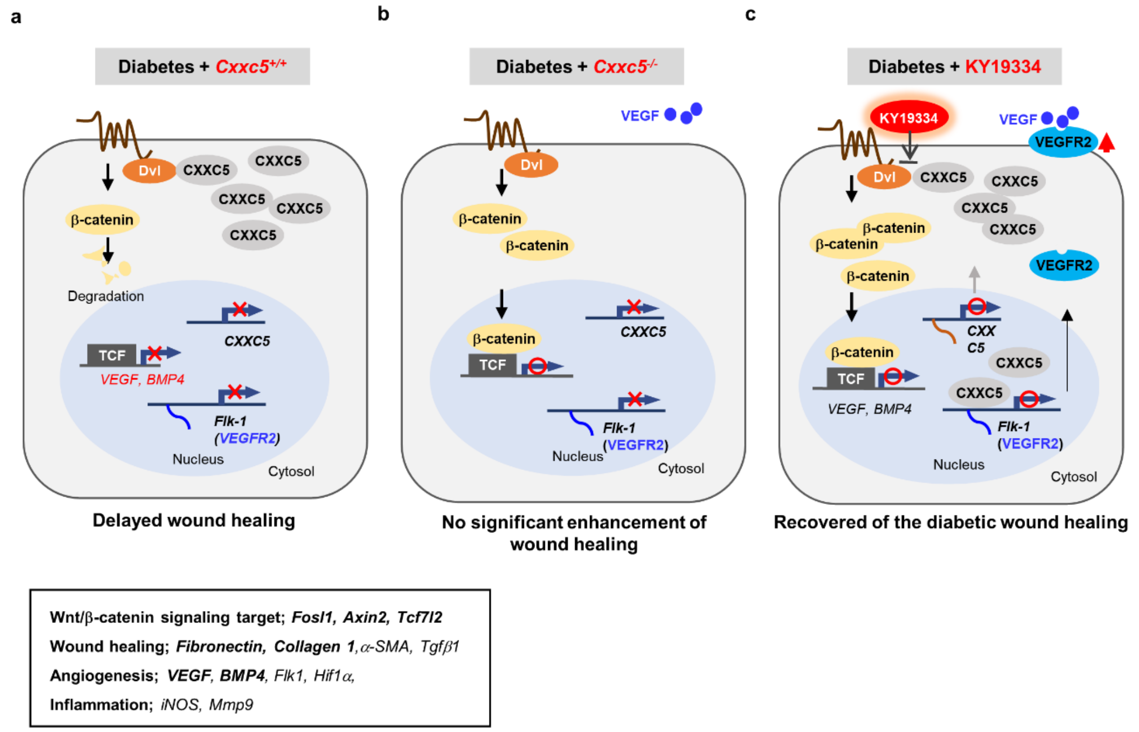

Supplementary Fig. 11 The different models of Wnt/β-catenin and VEGF signaling in genetic status of CXXC5 or KY19334. a, b Unlike the wild-type mice, diabetic wound healing was not significantly enhanced in the *Cxxc5*$^{-/-}$ mice because of the abolishment of the role of CXXC5 as a transcription factor for *Flk-1*, which encodes VEGFR2 **a** *vs* **b**. **c** The diabetic wound healing of *Cxxc5*$^{+/+}$ mice with KY19334 treatment was enhanced by both restorative activation of suppressed Wnt/β-catenin signaling and induction of VEGFR2 by transcriptional induction of *Flk-1* by nuclear CXXC5.

Supplementary Table. 1 Sequences of real-time PCR primers

Gene	Forward (5’- 3’)	Reverse (5’- 3’)
Axin2	TGGAGAGTGAGCGGCAGAGC	TGGAGACGAGCGGGCAGA
Fosl1	AACCGGAGGAAGGAACTGAC	CTGCAGCCCAGATTTCTCA
Tcf7l2	TGTGTACCCAATCACGACAGGAG	GATTCCGGTCGTGTGCAGAG
Fn1	AAGACCATACCTGCCGAATG	GAACATGACCGATTTGGACC
Acta2	GTCCCAGACATCAGGGAGTAA	TCGGATACTTCAGCGTCAGGA
Col1a1	CCTCAGGGTATTGCTGGACA	GAAGGACCTTGTTTGCCAGG
Tgfb1	TGACGTCACTGGAGTTGTACGG	GGTTCATGTCATGGATGGTGC
Flk1	GGCGGTGGTGACAGTATCTT	TCTCCGGCAAGCTCAAT
Vegfa	CAGGCTGCTGTAACGATGAA	AATGCTTTCTCCGCTCTGAA
Hif1a	GGTTCCAGCAGACCCAGTTA	AGGCTCCTTGGATGAGCTTT
Mmp9	AACATCTGGCACTCCACACC	GCAGAAGTTCTTTGGCCTGC
Nos2	TTCACCCAGTTGTGCATCGACCTA	TCCATGGTCACCTCCAACACAAGA

Supplementary Table. 2 Characteristics of patients with diabetic foot ulcer

**Demographic factors**	
Male gender, No. (%)	23 (88.46)
Age (years)	63.58 ± 11.14
BMI (kg/m2)	23.17 ± 3.56
**Medical status at screening**	
HbA1c (range)	8.07 ± 2.66
Blood glucose level	192 ± 154.58
**Baseline DFU characteristics**	
Location of DFU, No. (%)	
Toe	12 (46.15)
Sole	2 (7.69)
Dorsum	8 (30.77)
Ankle	4 (15.38)
Severity (Wagner grade ≥ 3), No. (%)	21 (80.77)
Chronic wound, No. (%)	19 (74.08)
**Complications/other diagnosis, besides T2DM and DFU, n (%)**	
PAOD	12 (46.15)
CAOD	10 (38.46)
HTN	20 (76.92)
CKD	3 (11.54)
ESRD on HD	10 (38.46)

Values are expressed as mean ± standard deviation for continuous variables. Categorical variables are expressed as the number and percent of patients with the measured characteristic.

Abbreviations: No., number of subjects with characteristic; BMI, Body mass index; HbA1c, Hemoglobin A1c; DFU, Diabetic foot ulcer; T2DM, Type 2 diabetes mellitus; PAOD, Peripheral arterial occlusive disease; CAOD, Coronary arterial occlusive disease; HTN, Hypertension; CKD, Chronic kidney disease; ESRD on HD, End stage renal disease on hemodialysis.
